# Supplementary material for: Disuse-associated loss of the protease LONP1 in muscle impairs mitochondrial function and causes reduced skeletal muscle mass and strength
Source: Nat Commun. 2022 Feb 16;13:894. doi: 10.1038/s41467-022-28557-5 (PMC8850466; doi:10.1038/s41467-022-28557-5)

Supplementary Information

Disuse-associated loss of the protease LONP1 in muscle impairs mitochondrial function and causes reduced skeletal muscle mass and strength

Zhisheng Xu[1,#], Tingting Fu[1,#], Qiqi Guo[1,#], Danxia Zhou[1], Wanping Sun[1], Zheng Zhou[1], Xinyi Chen[1], Jingzi Zhang[2], Lin Liu[1], Liwei Xiao[1], Yujing Yin[1], Yuhuan Jia[1], Erkai Pang[3], Yuncong Chen[4], Xin Pan[5], Lei Fang[2], Min-sheng Zhu[6], Wenyong Fei[3], Bin Lu[7] and Zhenji Gan[1,8,9,*]

[1]The State Key Laboratory of Pharmaceutical Biotechnology and MOE Key Laboratory of Model Animal for Disease Study, Model Animal Research Center, Department of Spine Surgery, Nanjing Drum Tower Hospital, The Affiliated Hospital of Nanjing University Medical School, Nanjing University Medical School, Nanjing University, Nanjing, China.
[2]Jiangsu Key Laboratory of Molecular Medicine & Chemistry and Biomedicine Innovation Center, Medical School of Nanjing University, Nanjing, China.
[3]Sports Medicine Department, Northern Jiangsu People's Hospital, Clinical Medical College, Yangzhou University, Yangzhou, China.
[4]State Key Laboratory of Coordination Chemistry, School of Chemistry and Chemical Engineering, Chemistry and Biomedicine Innovation Center (ChemBIC), Nanjing University, Nanjing, China.
[5]State Key Laboratory of Proteomics, Institute of Basic Medical Sciences, National Center of Biomedical Analysis, Beijing, China.
[6]The State Key Laboratory of Pharmaceutical Biotechnology and MOE Key Laboratory of Model Animal for Disease Study, Model Animal Research Center, Nanjing University Medical School, Nanjing University, Nanjing, China.
[7]Department of Biochemistry and Molecular Biology, School of Basic Medical Sciences, Hengyang Medical School, University of South China, Hengyang, China
[8]Jiangsu Key Laboratory of Molecular Medicine, Nanjing University Medical School, Nanjing University, Nanjing, China.
[9]Chemistry and Biomedicine Innovation Center (ChemBIC), Nanjing University, Nanjing, China.

[#]These authors contributed equally
[*]To whom to address correspondence:
Zhenji Gan, Ph.D.
ganzj@nju.edu.cn

Contents:

1. Supplementary Figures 1-9;
2. Supplementary Tables 1-2;
3. Uncropped scans for Supplementary Figures 1-9.

Supplementary Figure 1

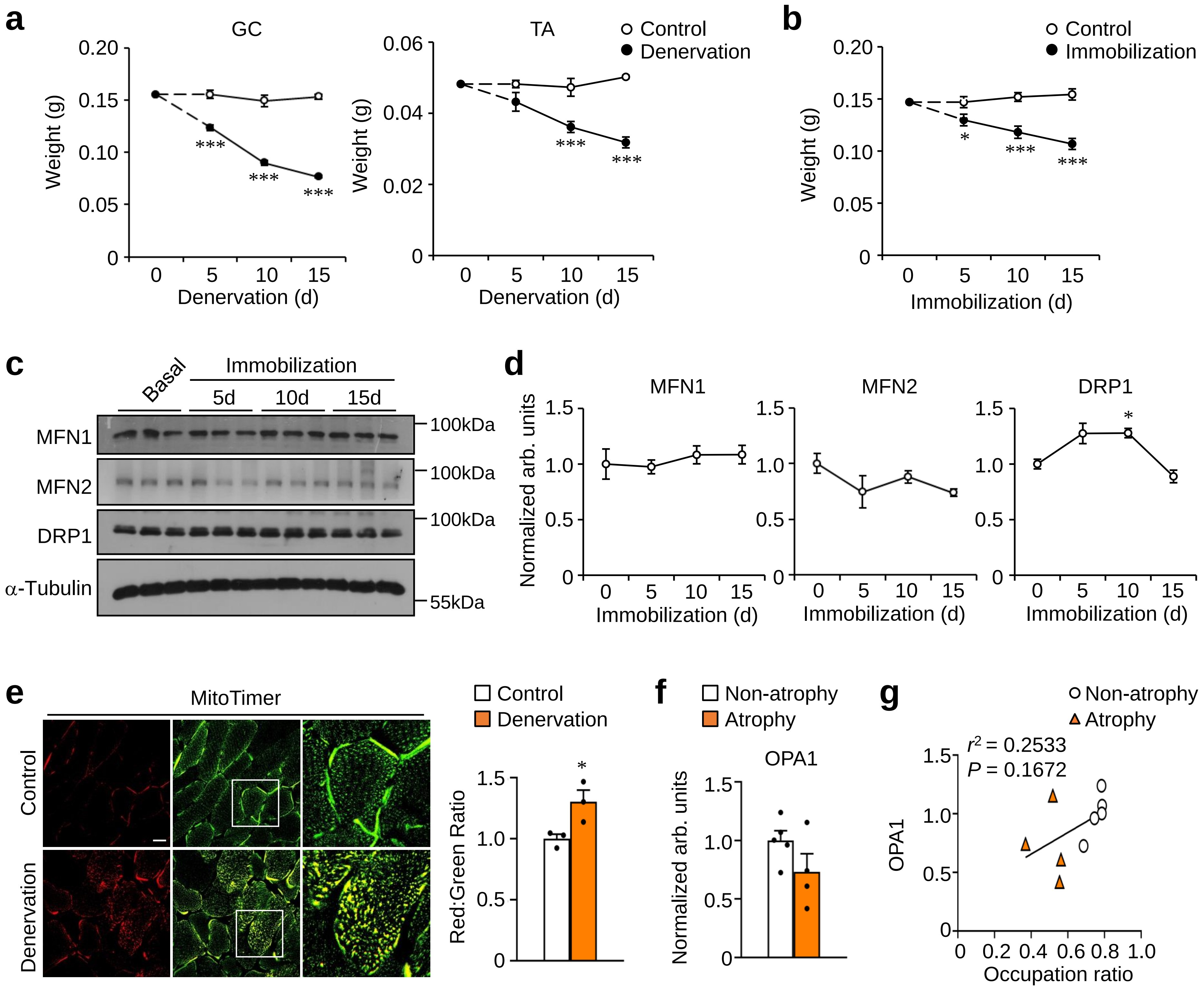

Supplementary Figure 1. Disuse-related muscle loss in mice is associated with decreased mitochondrial LONP1 protein. (**a**) The gastrocnemius (GC) and tibialis anterior (TA) muscle weight from non-denervated (Control) and denervated (Denervation) limbs of WT mice for the indicated number of days (d). n = 4 mice per group. (**b**) The GC muscle weight from non-immobilized (Control) and immobilized (Immobilization) limbs of WT mice for the indicated number of days (d). n = 7-8 mice per group. (**c**) Representative western blot analysis performed with GC muscle total protein extracts prepared from non-immobilized (basal) and immobilized limbs of WT mice for the indicated number of days (d) using indicated antibodies. (**d**) Quantification of MFN1/Tubulin, MFN2/Tubulin and DRP1/Tubulin signal ratios normalized (= 1.0) to basal is shown. n = 3 mice per group. (**e**) (Left) Representative confocal images of TA muscles in HSA-Cre/MitoTimer mice at 5 days post denervation. The scale bar represents 20 μm. (Right) Quantification of MitoTimer Red:Green ratio normalized (= 1.0) is shown. n = 3 mice per group. (**f**) Quantification of OPA1/Tubulin data shown in (Fig. 1g). n = 4-5 patients per group. (**g**) Correlation between the protein expression of OPA1 and the occupation ratios. n = 9 patients. Pearson's correlation analysis was used to determine the correlation. Values represent mean ± SEM; for (**a**), (**b**), (**e**) and (**f**), *P < 0.05, **P < 0.01, ***P < 0.001 versus corresponding controls determined by two-tailed unpaired Student's t test; for (**d**), *P < 0.05 versus corresponding controls determined by one-way ANOVA coupled to a Fisher's LSD post hoc test. Source data are provided as a Source Data file.

Supplementary Figure 2

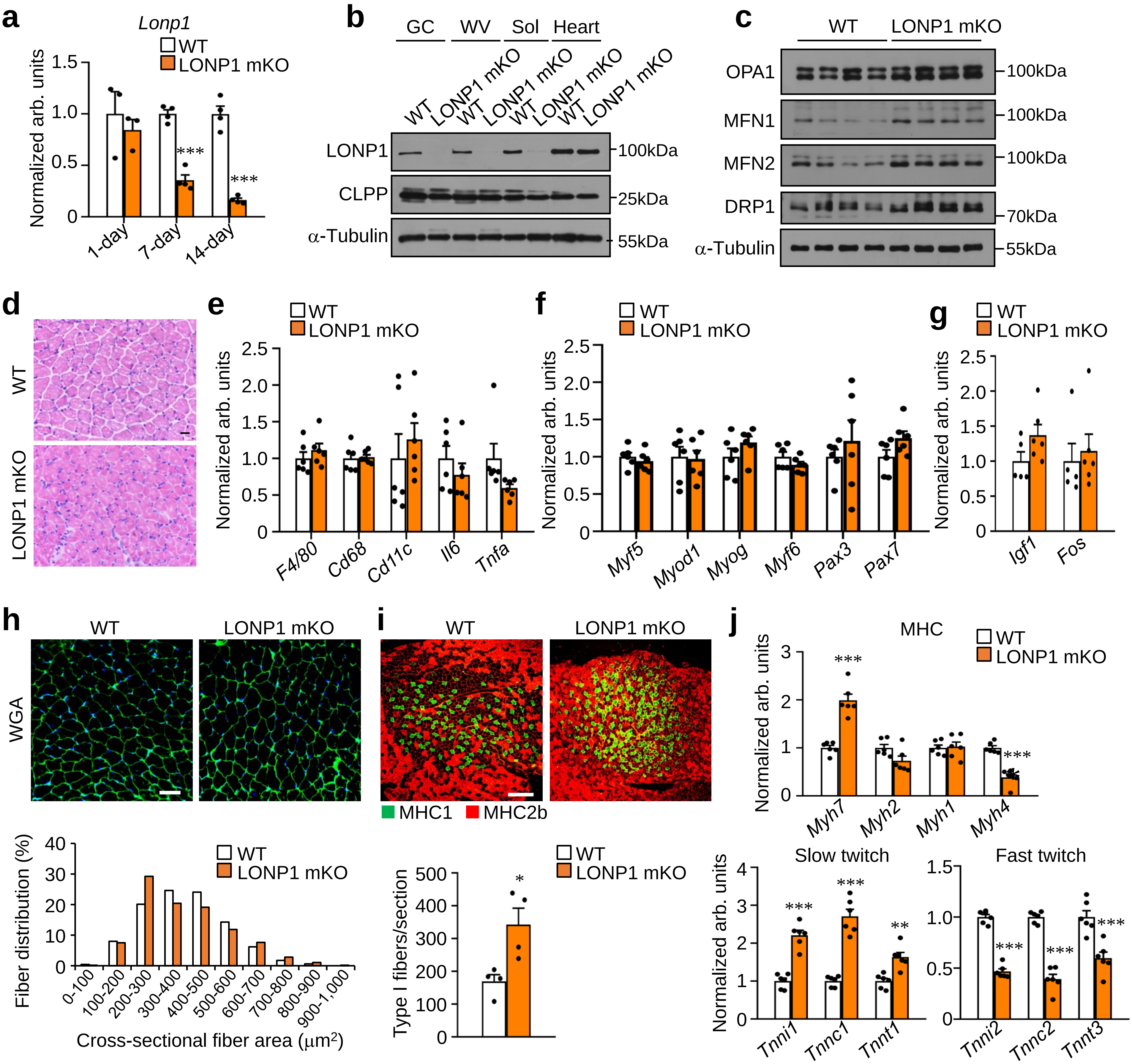

Supplementary Figure 2. Characterization of mice with skeletal muscle-specific ablation of the *Lonp1* gene. (**a**) RT-qPCR analysis of *Lonp1* expression in skeletal muscle from indicated genotypes at the age of 1, 7 and 14 days. n = 3-4 mice per group. (**b**) Western blot analysis of LONP1 and CLPP expression in GC, white vastus lateralis (WV), soleus (Sol) muscles and hearts of WT and LONP1 mKO mice. (**c**) Representative Western blot analysis performed with WV muscle total protein extracts prepared from WT and LONP1 mKO mice using indicated antibodies. n = 4 mice per group. (**d**) H&E staining of the GC muscle from 2-week-old WT and LONP1 mKO mice. Representative images were shown. The scale bar represents 20 μm. n = 4-6 mice per group. (**e**) RT-qPCR analysis of inflammation-related gene expression in GC muscles from 2-week-old indicated mice. n = 6 mice per group. (**f**) RT-qPCR analysis of key myogenic regulator gene expression in GC muscle from 2-week-old indicated mice. n = 6 mice per group. (**g**) Expression of *Igf1* and *Fos* genes in liver from 6-week-old indicated mice. n = 5-6 mice per group. (**h**) (Top) Representative WGA staining of GC muscle from indicated mice at the age of 2 weeks. Scale bar represents 50 μm. (Bottom) Cross-sectional areas of GC myofibers were measured by ImageJ. n = 6 mice per group. (**i**) (Top) Cross section of GC muscle from 6-week-old male LONP1 mKO and WT mice stained for MHC1 (green) and MHC2b (red). Scale bars: 250 μm. (Bottom) Quantification of IF data. n = 4 mice per group. (**j**) Expression of MHC and representative slow/fast-twitch troponin genes (RT-qPCR) in entire GC muscle from indicated mice. n = 6 mice per group. Values represent mean ± SEM; for (**a**), (**e**), (**f**), (**g**), (**i**) and (**j**), $*P < 0.05$, $**P < 0.01$, $***P < 0.001$ versus WT controls determined by two-tailed unpaired Student's *t* test. Source data are provided as a Source Data file.

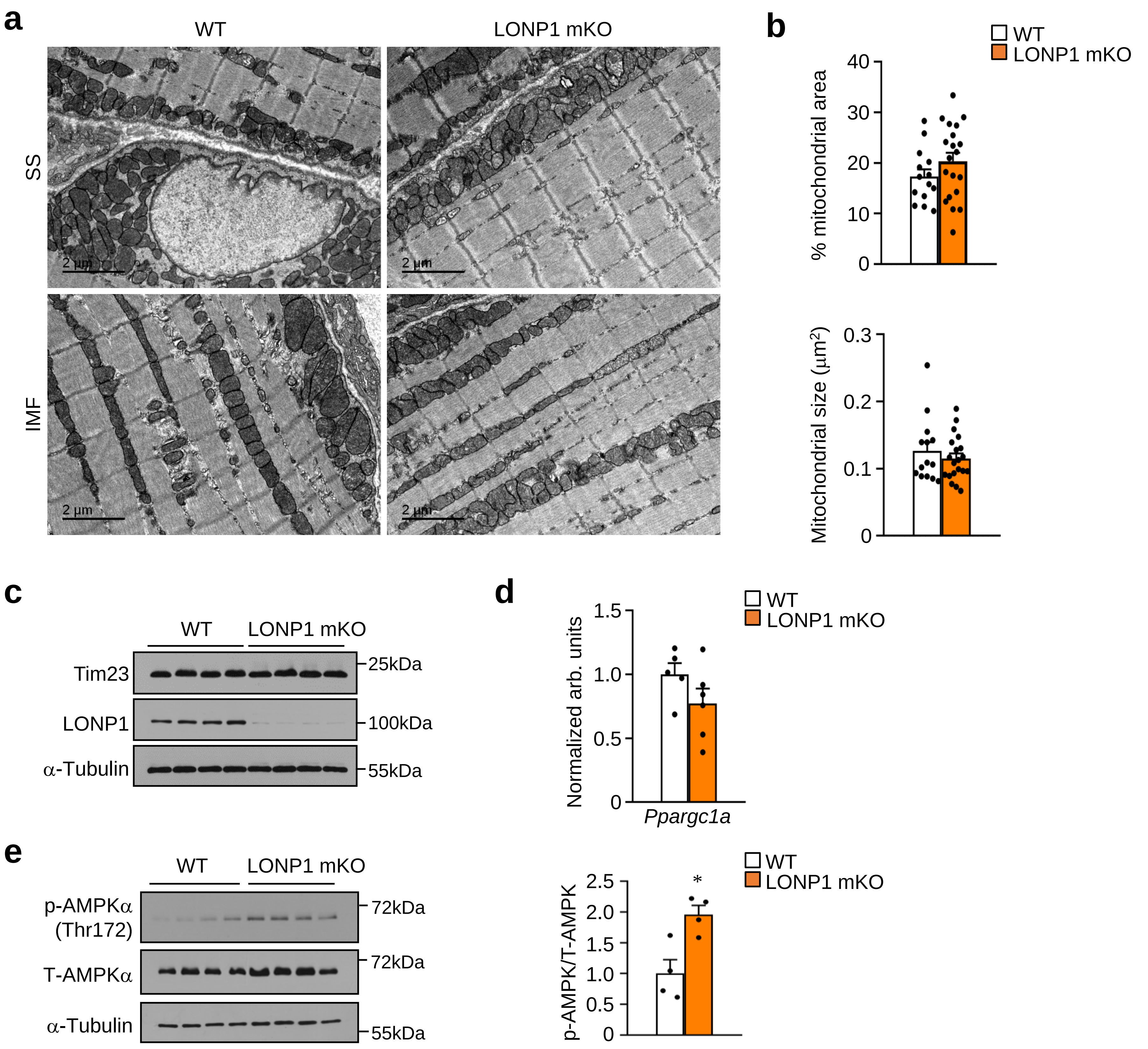

Supplementary Figure 3. No change in mitochondrial biogenesis in LONP1 mKO muscle. (**a**) Representative electron micrographs of the soleus muscle showing subsarcolemmal (SS) and intermyofibrillar (IMF) mitochondria in sections from 6-week-old WT and LONP1 mKO mice. n = 4-6 mice per group. The scale bar represents 2 μm. (**b**) Percentage of mitochondrial area per muscle fiber area and average mitochondrial size were quantified from the indicated genotypes. n = 3 mice per group. (**c**) Western blot analysis performed with WV muscle total protein extracts prepared from the 6-week-old indicated mice using Tim23, LONP1 and α-Tubulin (control) antibodies. n = 4 mice per group. (**d**) Expression of *Ppargc1a* gene (RT-qPCR) in GC muscle from the indicated mice. n = 5-6 mice per group. (**e**) (Left) Representative Western blot analysis performed with WV muscle total protein extracts prepared from 6-week-old WT and LONP1 mKO mice using indicated antibodies. (Right) Quantification of the p-AMPK/T-AMPK signal ratios normalized (= 1.0) to WT controls is shown. n =4 mice per group. Values represent mean ± SEM; for (**b**), (**d**) and (**e**) $*P < 0.05$ versus WT controls determined by two-tailed unpaired Student's *t* test. Source data are provided as a Source Data file.

Supplementary Figure 4

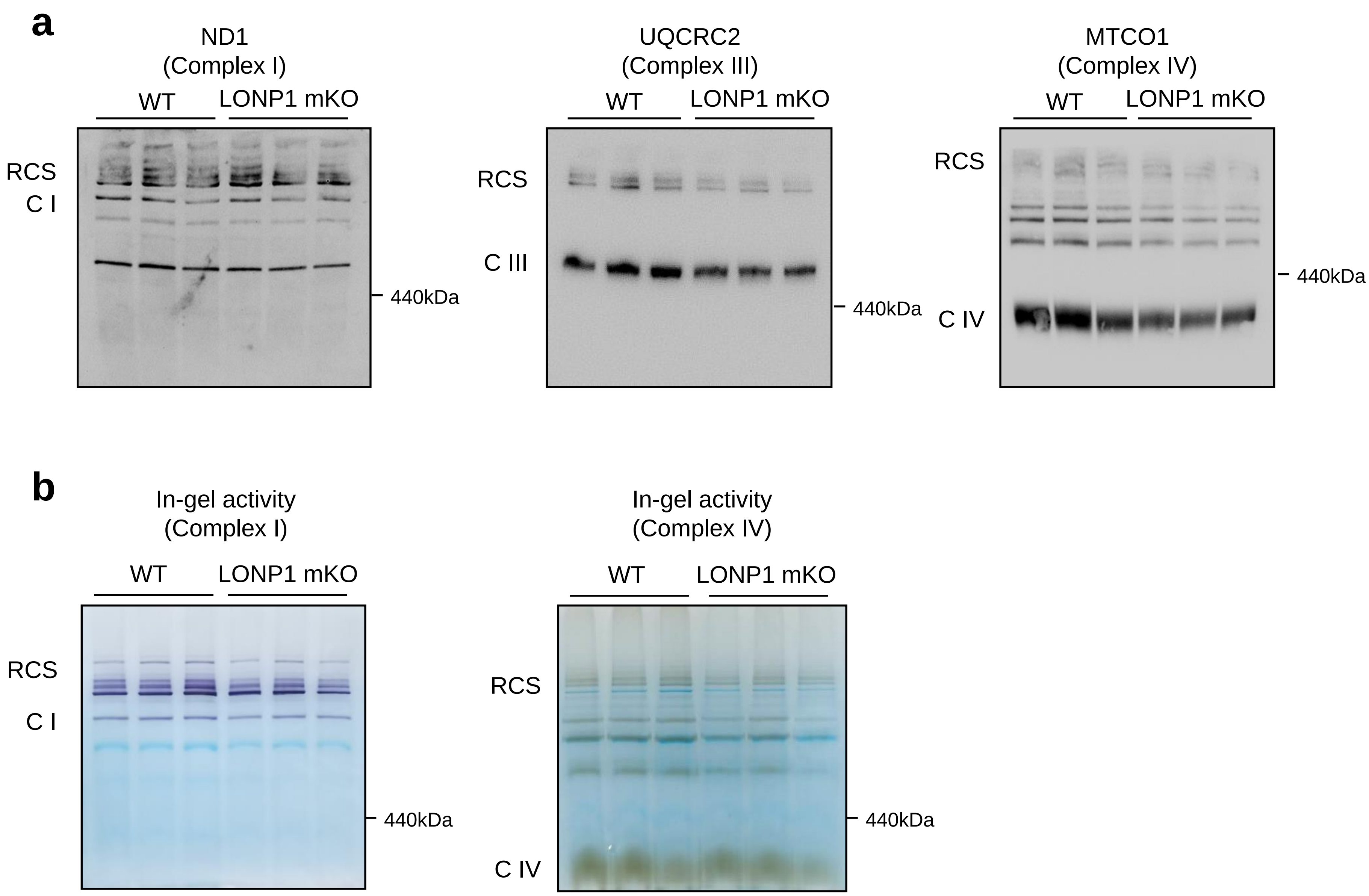

Supplementary Figure 4. Analysis of mitochondrial respiratory chain complexes in LONP1 mKO muscle. (**a**) Blue native polyacrylamide gel electrophoresis (BN-PAGE) analyses of the mitochondrial respiratory chain complexes followed by Western blot analysis using antibodies against C I (anti-ND1), C III (anti-UQCRC2) and C IV (anti-MTCO1). n = 3 mice per group. (**b**) In-gel activity of complex I and IV in skeletal muscle mitochondria performed after BN-PAGE. n = 3 mice per group. Source data are provided as a Source Data file.

Supplementary Figure 5

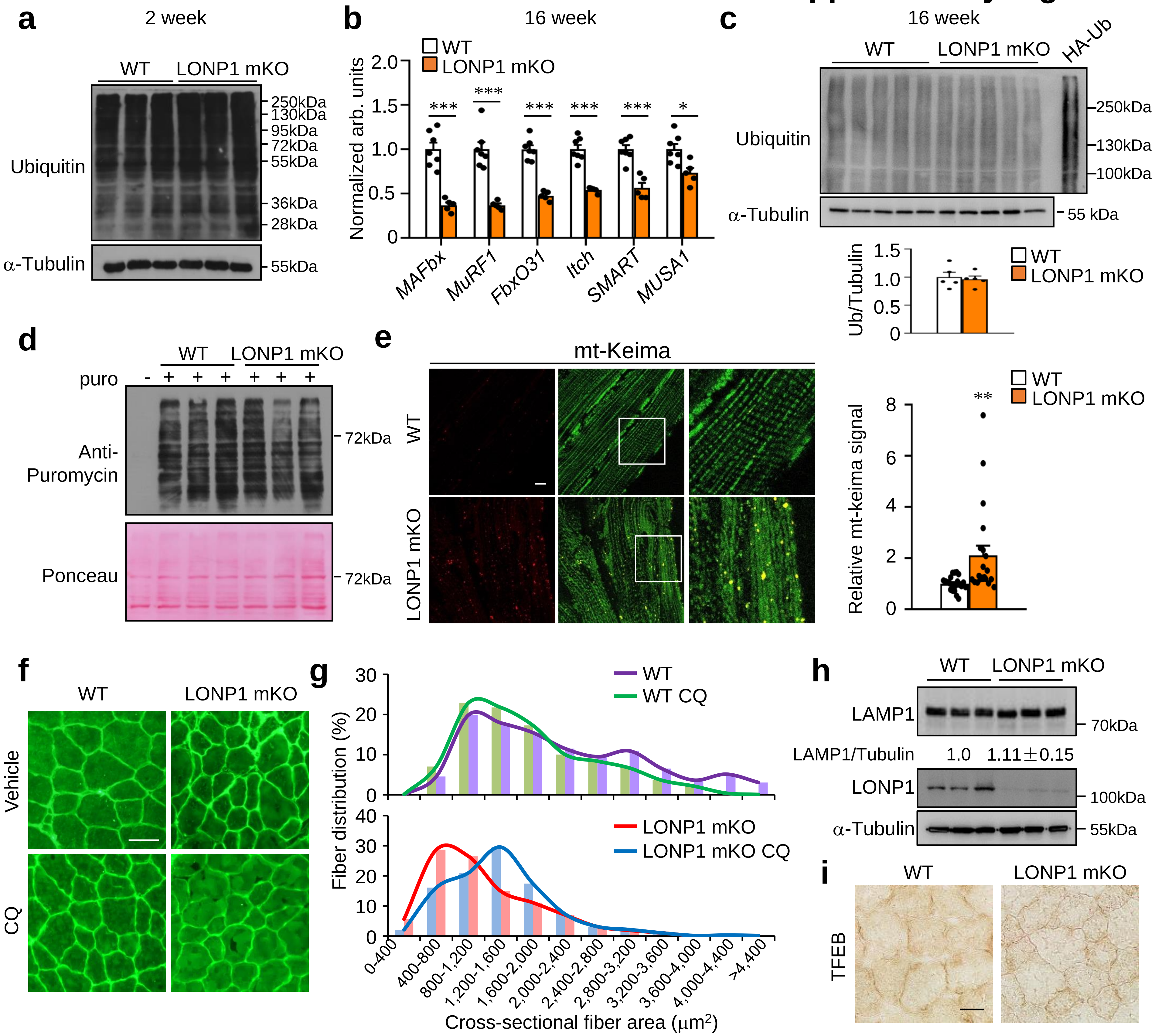

Supplementary Figure 5. Muscle LONP1 ablation does not affect ubiquitin-mediated protein degradation and protein synthesis but activates autophagy. (**a**) Representative Western blot analysis of poly-ubiquitinated proteins in WV muscle total protein extracts from 2-week-old WT and LONP1 mKO mice. n = 5 mice per group. (**b**) Expression of muscle atrophy-related genes (RT-qPCR) in GC muscles from indicated mice at the age of 16 weeks. n = 5-7 mice per group. (**c**) (Top) Representative Western blot analysis of ubiquitinated proteins in WV muscle total protein extracts from 16-week-old WT and LONP1 mKO mice. (Bottom) Quantification of the ubiquitinated proteins. n = 5 mice per group. (**d**) Representative Western blot analysis of puromycin incorporation in skeletal muscles from indicated mice at the age of 2 weeks. *In vivo* protein synthesis rates were measured via the SUnSET technique. n = 3 mice per group. (**e**) (Left) Representative confocal images of muscles from 2-week-old mt-Keima-TG mice and LONP1 mKO/mt-Keima mice. The scale bar represents 10 μm. The emission signal obtained after excitation with the 458-nm laser is shown in green, and that obtained after excitation with the 561-nm laser is shown in red. (Right) Quantification of the red/green mt-Keima signal ratios normalized (= 1.0) to the WT controls is shown. n = 3 mice per group. (**f**) Representative WGA staining of TA muscles from chloroquine (CQ)-treated and untreated WT and LONP1 mKO mice. Scale bar represents 50 μm. n = 4-6 mice per group. (**g**) Quantification of fiber size distribution in TA muscles from indicated mice. n = 4-6 mice per group. (**h**) Representative Western blot analysis performed with WV muscle total protein extracts prepared from 2-week-old WT and LONP1 mKO mice using indicated antibodies. Quantification of the LAMP1/Tubulin signal ratios were normalized (= 1.0) to WT controls and presented below the corresponding bands. n = 3 mice per group. (**i**) TFEB immunohistochemical analysis of LONP1 mKO muscles. GC muscle cryosections were immunostained by using anti-TFEB antibody. Scale bar represents 20 μm. n = 4 mice per group. Values represent mean ± SEM; for (**b**), (**c**) and (**e**), *$P < 0.05$, **$P < 0.01$, ***$P < 0.001$ versus corresponding WT controls determined by two-tailed unpaired Student's *t* test. Source data are provided as a Source Data file.

Supplementary Figure 6

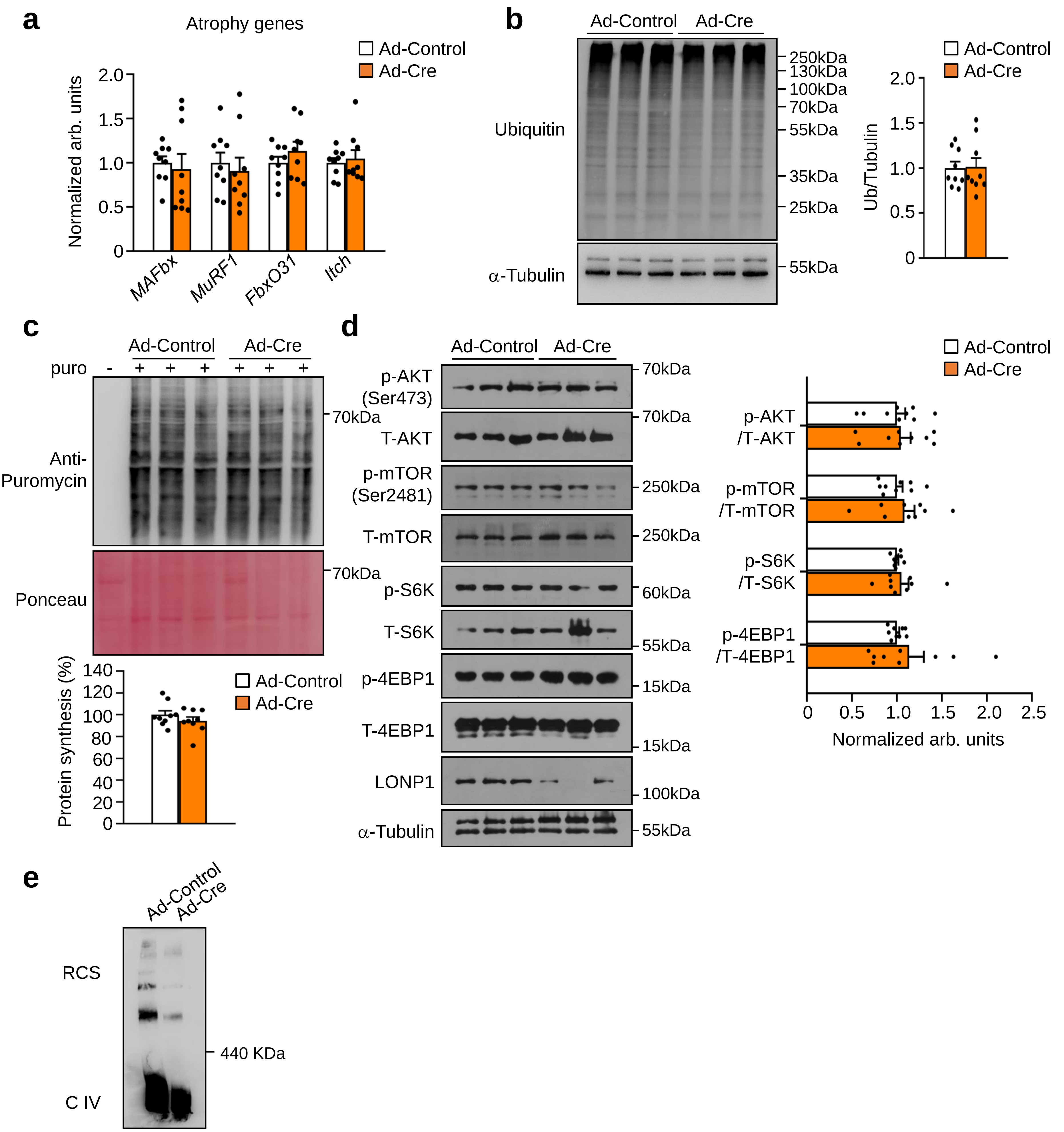

Supplementary Figure 6. LONP1 deficiency in primary myotubes does not affect ubiquitin-mediated protein degradation and protein synthesis. Primary myoblasts isolated from *Lonp1*$^{f/f}$ mice were infected with an adenovirus overexpressing Cre or control virus, followed by differentiation into myotubes. (**a**) Expression of muscle atrophy-related genes (RT-qPCR) in indicated myotubes. n = 3 independent experiments. (**b**) (Left) Representative Western blot analysis of poly-ubiquitinated proteins in indicated myotubes. (Right) Quantification of the ubiquitinated proteins. n = 3 independent experiments. (**c**) (Top) Representative Western blot analysis of puromycin incorporation in indicated myotubes. (Bottom) Quantification of puromycin incorporation. n = 3 independent experiments. (**d**) (Left) Representative Western blot analysis of total protein extracts in myotubes using the indicated antibodies. (Right) Quantification of the p-AKT/AKT, p-mTOR/mTOR, p-S6K/S6K and p-4EBP1/4EBP1 signal ratios normalized (= 1.0) to controls is shown. n = 3 independent experiments. (**e**) Representative BN-PAGE analyses of the mitochondrial respiratory chain complexes followed by Western blot analysis using antibodies against C IV (anti-MTCO1). n = 3 independent experiments. Values represent mean ± SEM; for (**a-d**), two-tailed unpaired Student's *t* test. Source data are provided as a Source Data file.

Supplementary Figure 7

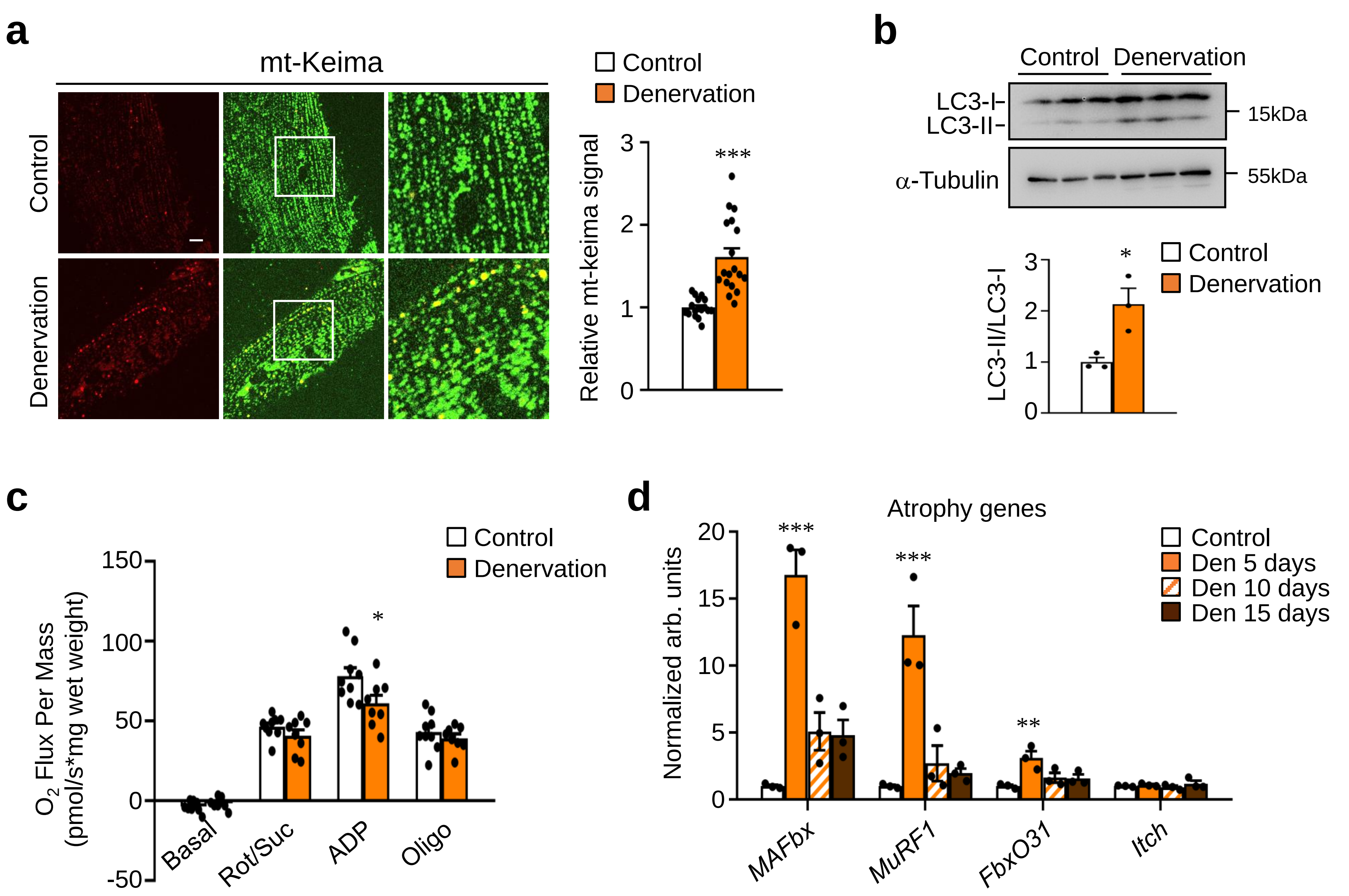

Supplementary Figure 7. Denervation-induced muscle disuse leads to suppressed expression of LONP1 with increased muscle autophagy and mitochondrial dysfunction. (**a**) (Left) Representative confocal images of EDL muscles in mt-Keima Tg mice at 5 days post denervation. The scale bar represents 10 µm. (Right) Quantification of the red/green mt-Keima signal ratios normalized (= 1.0) to the controls is shown. n = 3 mice per group. (**b**) (Top) Representative Western blot analysis of GC muscles lysates from WT mice at 5 days post denervation. (Bottom) Quantification of the LC3-II/LC3-I signal ratios normalized (= 1.0) to non-denervated controls is shown. n = 3 mice per group. (**c**) Mitochondrial respiration rates were determined on mitochondria isolated from WT muscles at 5 days post denervation using succinate as substrates. Succinate/rotenone (Suc/Rot)-stimulated, ADP-dependent respiration, and oligomycin (oligo)-induced are shown. n = 8-9 mice per group. (**d**) Expression of muscle atrophy-related genes (RT-qPCR) in GC muscles from non-denervated (Control) and denervated (Den) limbs of WT mice for the indicated number of days. n = 3 mice per group. Values represent mean ± SEM; for (**a-c**), **P* < 0.05, ****P* < 0.001 versus controls determined by two-tailed unpaired Student's *t* test; for (**d**), ***P* < 0.01, ****P* < 0.001 versus controls determined by one-way ANOVA coupled to a Fisher's LSD post hoc test. Source data are provided as a Source Data file.

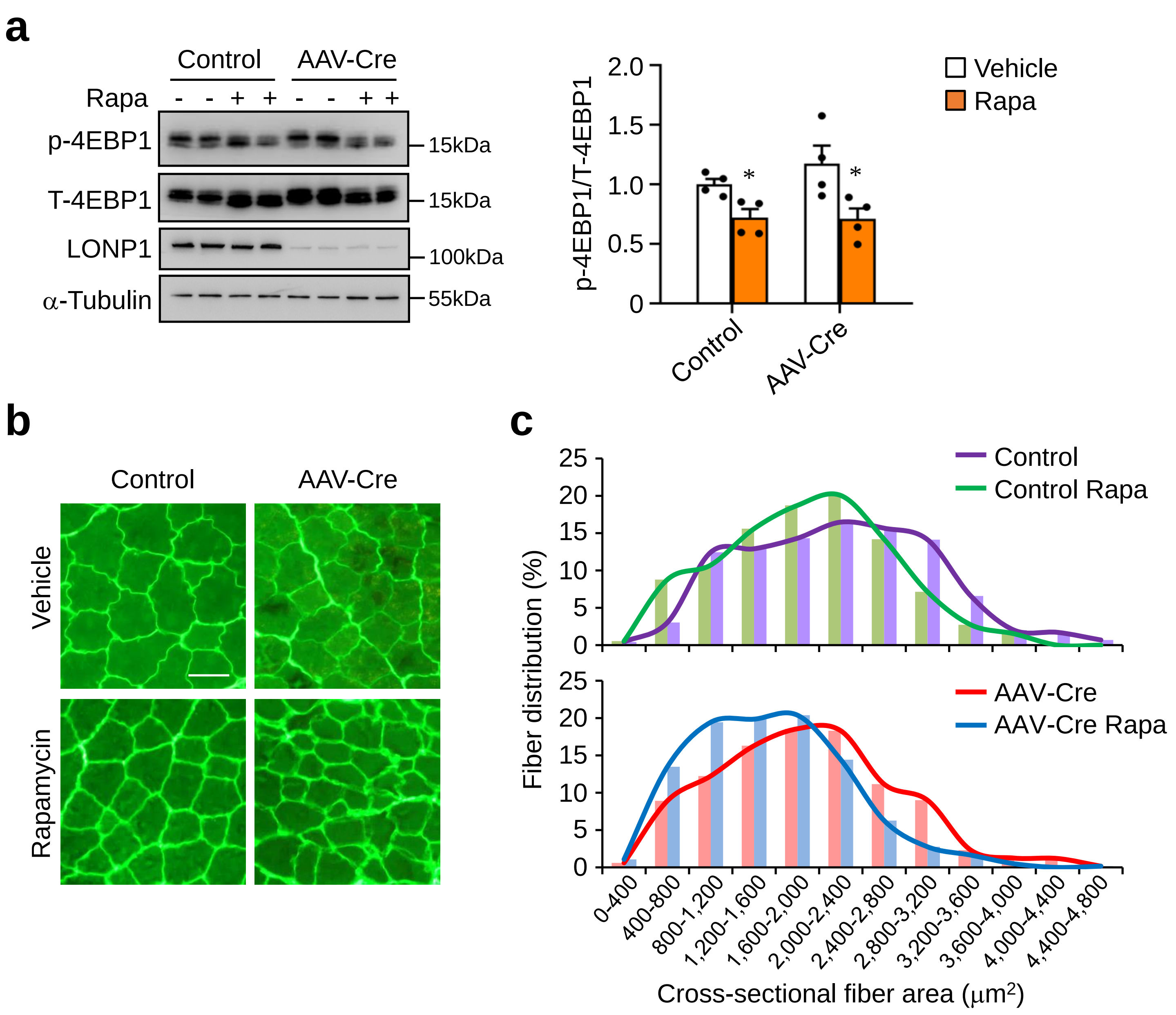

Supplementary Figure 8. Effects of rapamycin on LONP1-mediated regulation of muscle function. (**a-c**) 4 weeks after the injection of AAV-Cre or control viruses, *Lonp1*$^{f/f}$ mice were treated with vehicle or rapamycin (Rapa) as indicated for additional 4 weeks. (**a**) (Left) Representative Western blot analysis performed with TA muscle total protein extracts prepared from indicated mice using p-4EBP1, 4EBP1, LONP1 and α-Tubulin (control) antibodies. (Right) Quantification of the p-4EBP1/T-4EBP1 signal ratios. n = 4 mice per group. (**b**) Representative WGA staining of EDL muscles from indicated mice. Scale bar represents 50 µm. n = 5-6 mice per group. (**c**) Quantification of fiber size distribution in EDL muscles from indicated mice. n = 5-6 mice per group. Values represent mean ± SEM; for (**a**), *P < 0.05 versus corresponding controls determined by two-tailed unpaired Student's t test. Source data are provided as a Source Data file.

Supplementary Figure 9

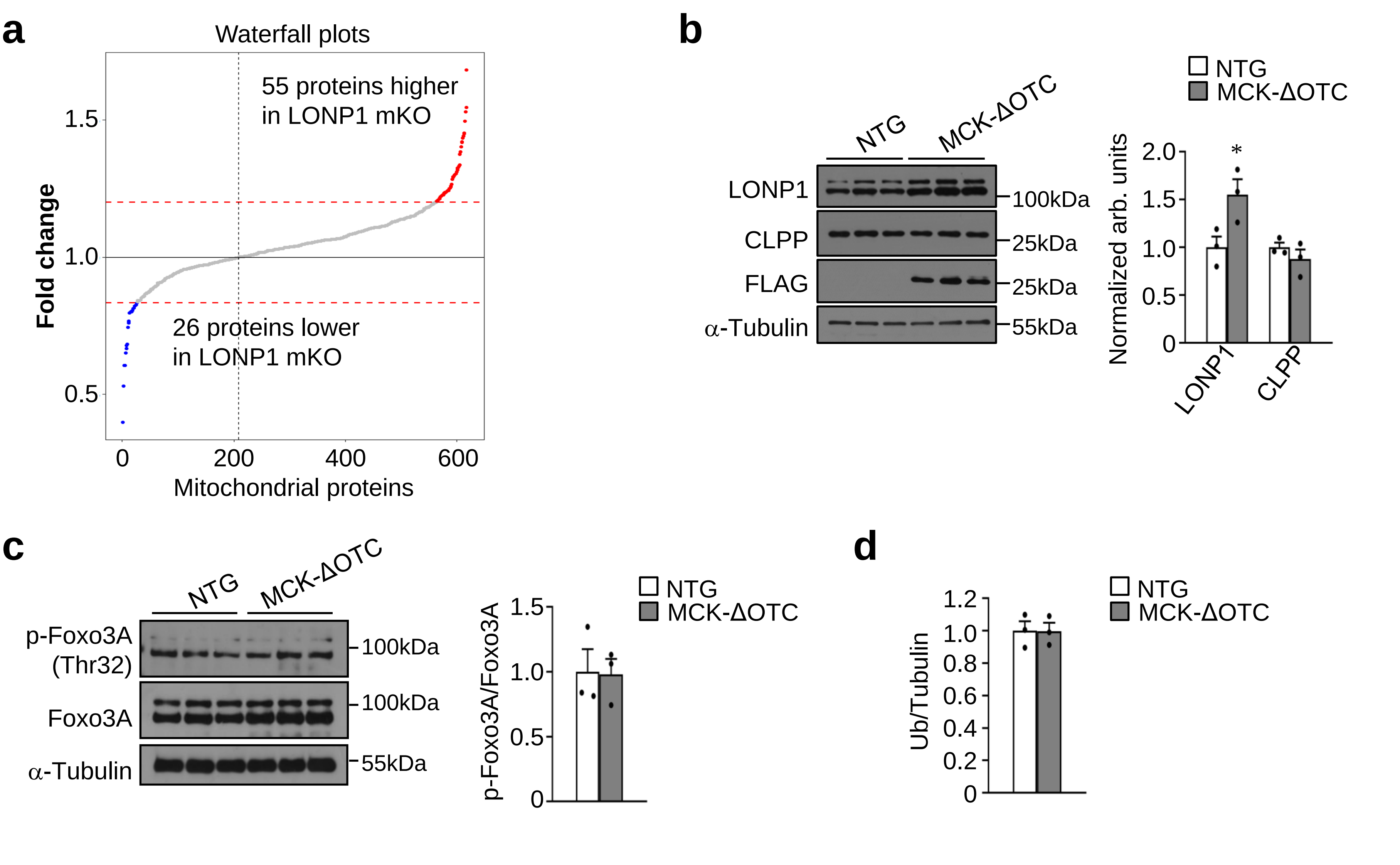

Supplementary Figure 9. LONP1 preserves mitochondrial proteostasis in skeletal muscle. (**a**) Waterfall plot the mass spectrometry proteomics data generated from mitochondria of LONP1 mKO compared to WT controls. (**b**) (Left) Representative Western blot analysis of LONP1 and CLPP protein expression in WV muscles from NTG and MCK-ΔOTC mice. (Right) Quantification of the LONP1/Tubulin and CLPP/Tubulin signal ratios normalized (= 1.0) to NTG mice is shown. n = 3 mice per group. (**c**) (Left) Representative Western blot analysis performed with WV muscle total protein extracts prepared from the indicated mice using indicated antibodies. (Right) Quantification of the p-Foxo3A/Foxo3A signal ratios normalized (= 1.0) to NTG control is shown. n = 3 mice per group. (**d**) Quantification of the ubiquitinated proteins of muscle total protein extracts from indicated mice. n = 3 mice per group. Values represent mean ± SEM; for (**b-d**), *$P < 0.05$ versus corresponding NTG controls determined by two-tailed unpaired Student's t test. Source data are provided as a Source Data file.

**Supplementary Table 1. Human Subject Characteristics**			
Group	Non-atrophy	Atrophy	*P* Value
Sex	n = 5 male	n = 4 male	
Age (yr)	52.6 ± 6.5	57.8 ± 12.5	0.6451
Height (cm)	167.0 ± 3.5	169.5 ± 1.0	0.5624
Weight (kg)	72.6 ± 4.1	77.3 ± 5.0	0.4900
Occupation Ratio	0.76 ± 0.02	0.50 ± 0.06	0.0008

Occupation Ratio: the ratio between the surface of the cross-section of the muscle belly and that of the supraspinatus fossa. Data represents the mean ± SEM. Differences were analyzed using two-tailed unpaired Student's *t* test, with a statistically significant difference defined as $P < 0.05$.

**Supplementary Table 2. RT-PCR primers**		
Mouse gene	***Forward***	***Reverse***
36b4	*5'-ATCCCTGACGCACCGCCGTGA*	*5'-TGCATCTGCTTGGAGCCCACGT*
MAFbx	*5'-AGTGAGGACCGGCTACTGTG*	*5'-GATCAAACGCTTGCGAATCT*
MuRF1	*5'-TGACATCTACAAGCAGGAGTGC*	*5'-TCGTCTTCGTGTTCCTTGC*
Fbxo31	*5'-AGACATCTTCCACGAGCACA*	*5'-GGTAGGTCAGGCAGTTGTCG*
Itch	*5'-AACAACGCCTTAACCCTAAGAA*	*5'-CATGCCCAGCTTGTACTGTTAC*
SMART	*5'-CTTGAACCTCTACATGCACCAG*	*5'-AAGTGGCTTGGGAAGTTGAC*
MUSA1	*5'-TTTCAACTGTGAGACTGAATTGC*	*5'-TTTCTACCTTTTGGCAGAAATAAAG*
Myf5	*5'-CTGCTCTGAGCCCACCAG*	*5'-GACAGGGCTGTTACATTCAGG*
Myod1	*5'-AGCACTACAGTGGCGACTCA*	*5'-GGCCGCTGTAATCCATCAT*
Myog	*5'-CCTTGCTCAGCTCCCTCA*	*5'-TGGGAGTTGCATTCACTGG*
Myf6	*5'-AGGGGCCTCGTGATAACTG*	*5'-GGAAGAAAGGCGCTGAAGA*
Pax3	*5'-GCGAGAAAAAGGCTAAACACA*	*5'-CGGAGCCTTCATCTGACTG*
Pax7	*5'-AGGAGCTGGCACAGAGGA*	*5'-GCACGCCGGTTACTGAAC*
Ppargc1a	*5'-GGACATGTGCAGCCAAGACTCT*	*5'-CACTTCAATCCACCCAGAAAGCT*
Fos	*5'-ACCAGTGTCTACCCCTGGAC*	*5'-GCTGGGGAATGGTAGTAGGA*
Igf1	*5'-TGTGCTGCATCGCTGCTTAC*	*5'-ACTCTTCCACGATGCCACG*
Park7	*5'-ACGATGTGGTGGTTCTTCCAGG*	*5'-CTGCACAGATGGCAGCTATGAG*
Lonp1	*5'-CATTGCCTTGAACCCTCTC*	*5'-ATGTCGCTCAGGTAGATGG*
F4/80	*5'-TTTCCTCGCCTGCTTCTTC*	*5'-CCCCGTCTCTGTATTCAACC*
Cd68	*5'-GCAGCACAGTGGACATTCAT*	*5'-TTGCATTTCCACAGCAGAAG*
Cd11c	*5'-CAGAACTTCCCAACTGCACA*	*5'-TCTCTGAAGCTGGCTCATCA*
Il6	*5'-TACCACTTCACAAGTCGGAGGC*	*5'-CTGCAAGTGCATCATCGTTGTTC*
TNFa	*5'-GGTGCCTATGTCTCAGCCTCTT*	*5'-GCCATAGAACTGATGAGAGGGAG*

Myh7	*5’-GCCAACTATGCTGGAGCTGATGCCC*	*5’-GGTGCGTGGAGCGCAAGTTTGTCATAAG*
Myh2	*5’-GGCACAAACTGCTGAAGCAGAGGC*	*5’-GGTGCTCCTGAGGTTGGTCATCAGC*
Myh1	*5’-GGCAGCAGCAGCTGCGGAAGCAGAGTCTGG*	*5’-GAGTGCTCCTCAGATTGGTCATTAGC*
Myh4	*5’-GAGCTACTGGATGCCAGTGAGCGC*	*5’-CTGGACGATGTCTTCCATCTCTCC*
Tnni1	*5'-TGAAGCCAAATGCCTCCACAACAC*	*5'-ACACCTTGTGCTTAGAGCCCAGTA*
Tnnc1	*5'-TGGATCCACCAGCTGGAATCAGAA*	*5'-GCTGATGCGGTTGTAGAGCACATT*
Tnnt1	*5'-AGCTCATGAAGGACGGTGACAAGA*	*5'-AACCGTGCAAGACCAGCATCTACT*
Tnni2	*5'-AGCAGCAAGGAGCTGGAAGA*	*5'-ATGGCGTCGGCAGACATAC*
Tnnc2	*5'-CCATCATCGAGGAGGTGGAC*	*5'-CTTCCCCTTCGCATCCTCTT*
Tnnt3	*5'-AACTGGAGACTGACAAATTCGAGT*	*5'-GCTGTGCTTCTGGGTTTGGT*
Primers for quantification of mtDNA		
mt-Nd1	*5’-CCCATTCGCGTTATCTT*	*5’-AAGTTGATCGTAACGGAAGC*
Lpl	*5’-GATGGACGGTAAGAGTGATTC*	*5’-ATCCAAGGGTAGCAGACAGGT*

Uncropped scans for Supplementary Figure 1

Supplementary Figure 1c

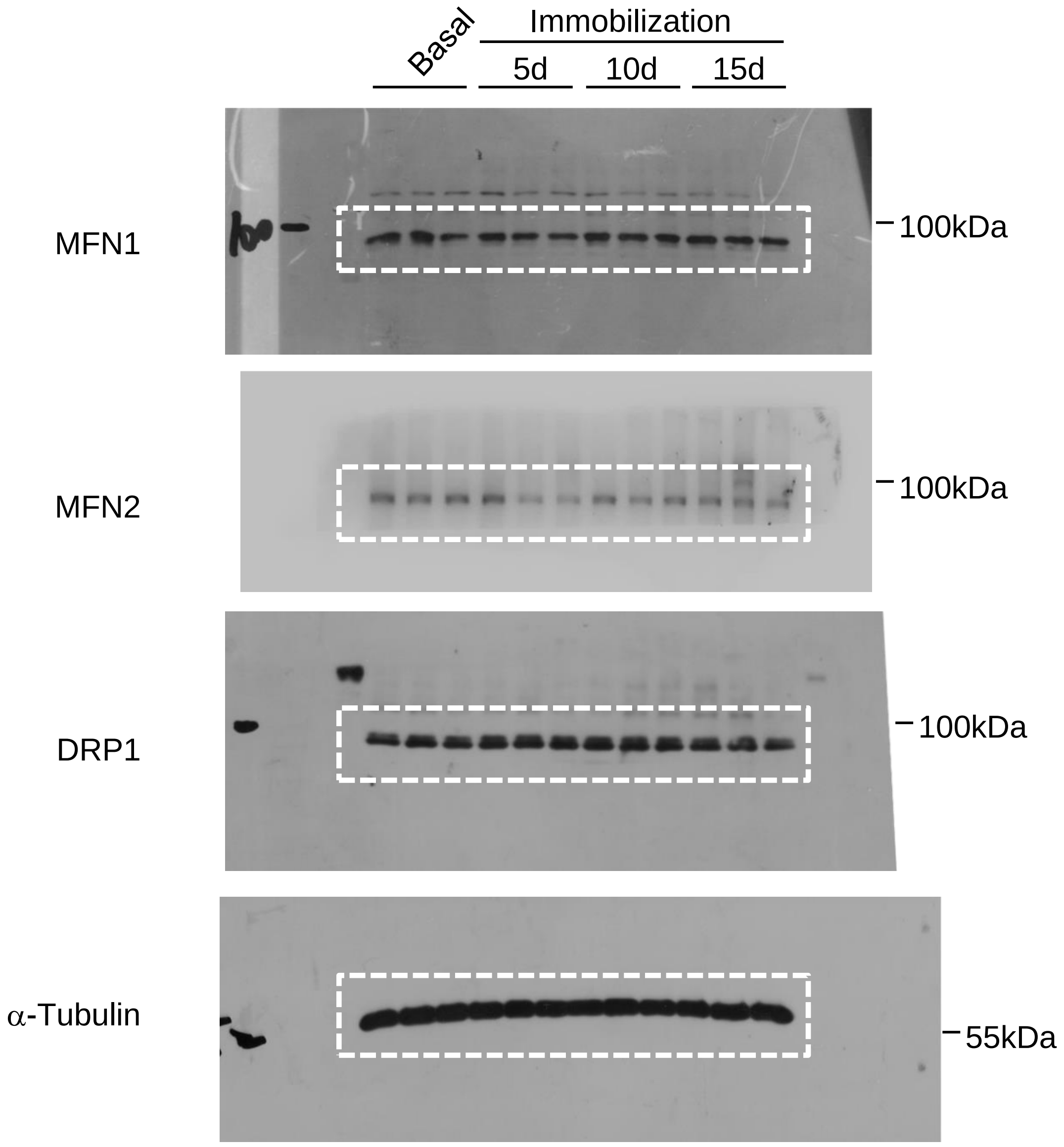

Uncropped scans for Supplementary Figure 2

Supplementary Figure 2b

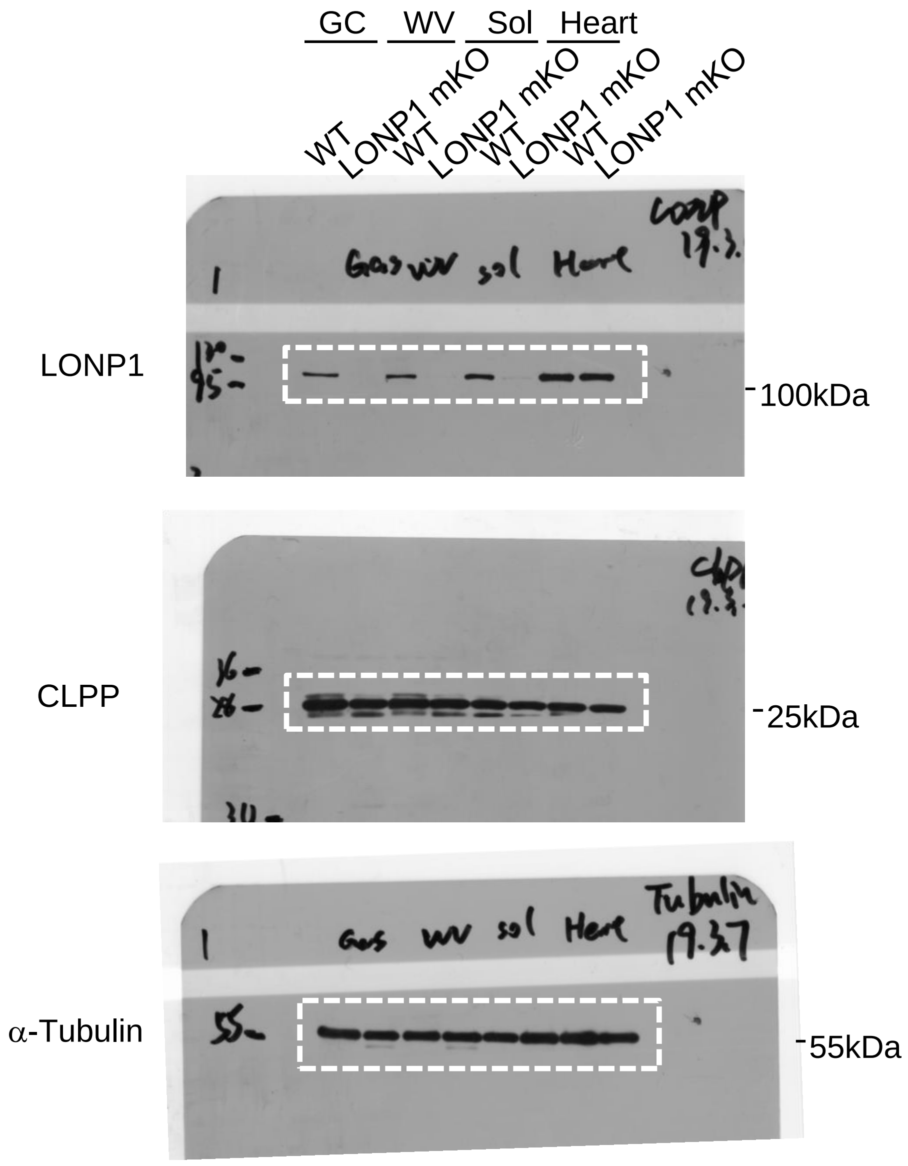

Supplementary Figure 2c

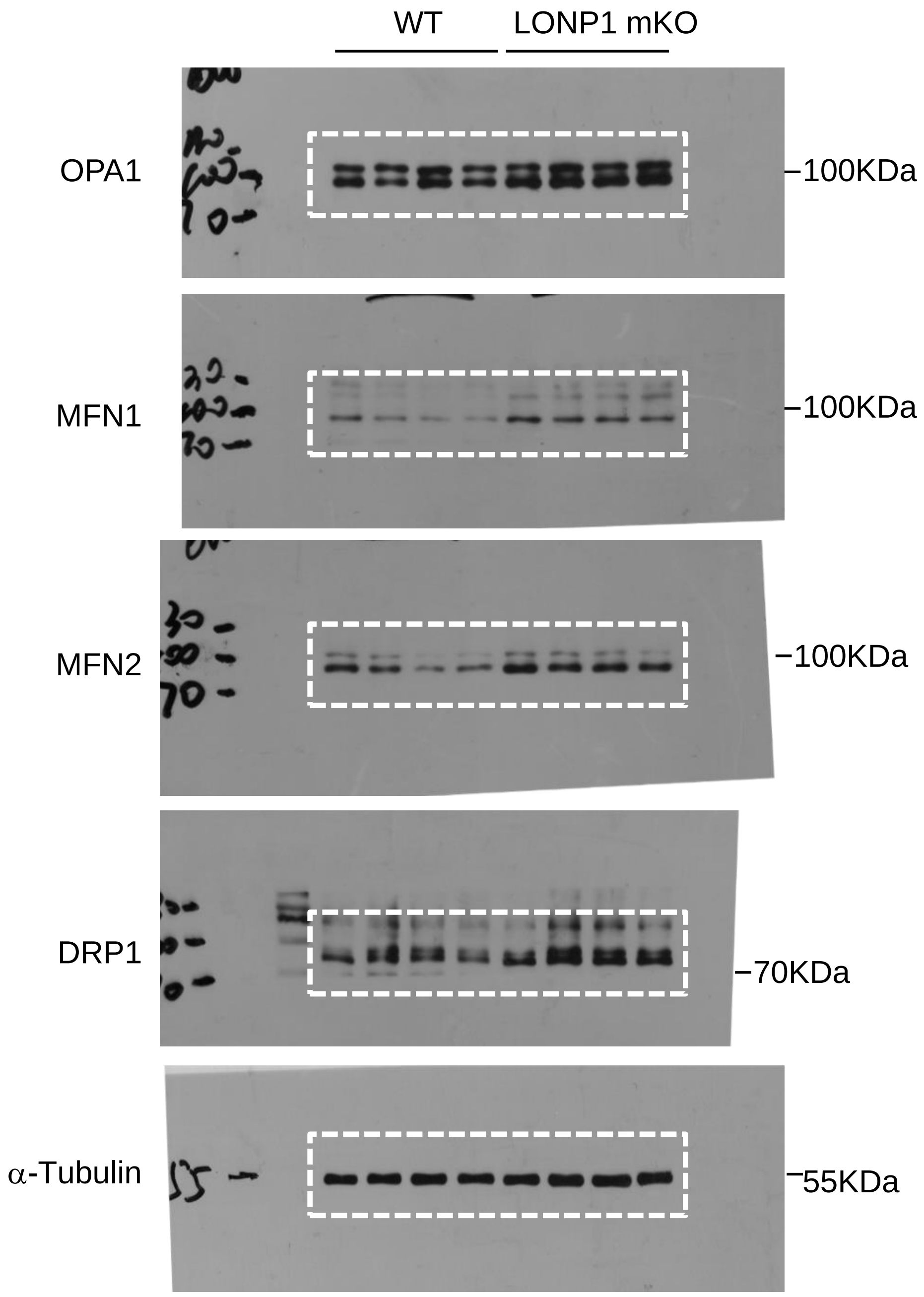

Uncropped scans for Supplementary Figure 3

Supplementary Figure 3c

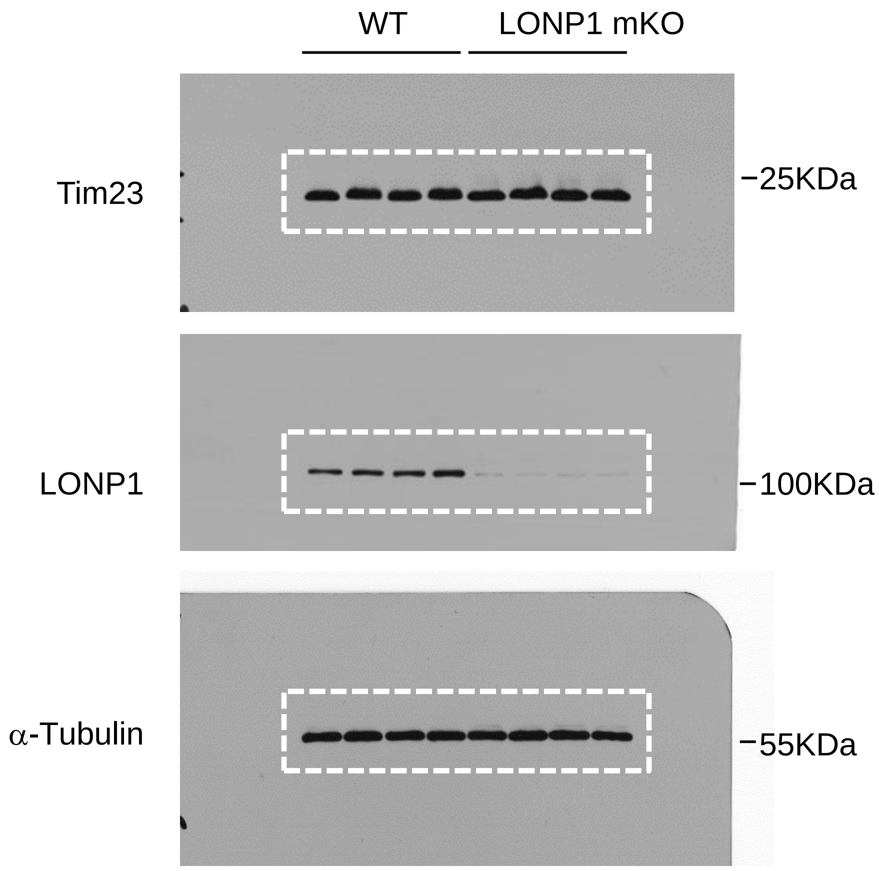

Supplementary Figure 3e

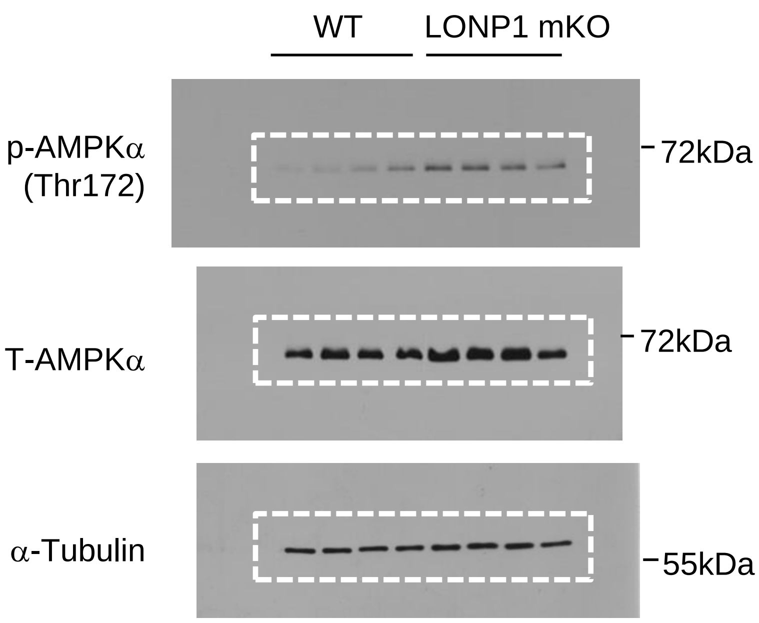

Uncropped scans for Supplementary Figure 4

Supplementary Figure 4a

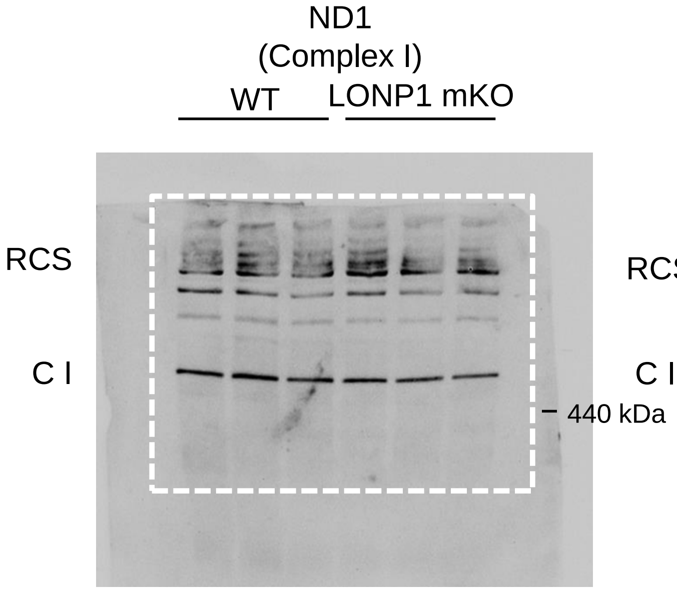

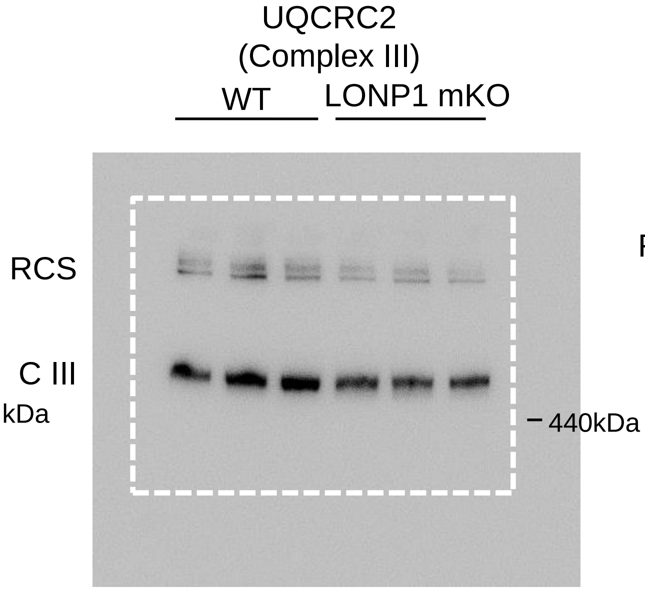

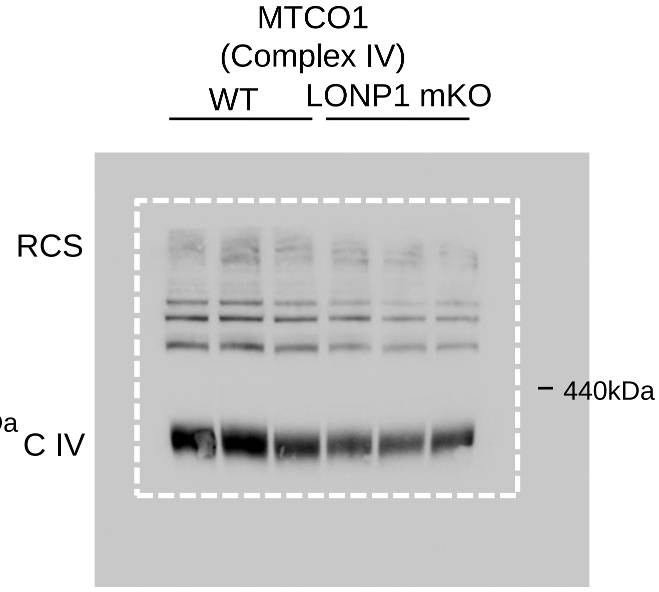

Supplementary Figure 4b

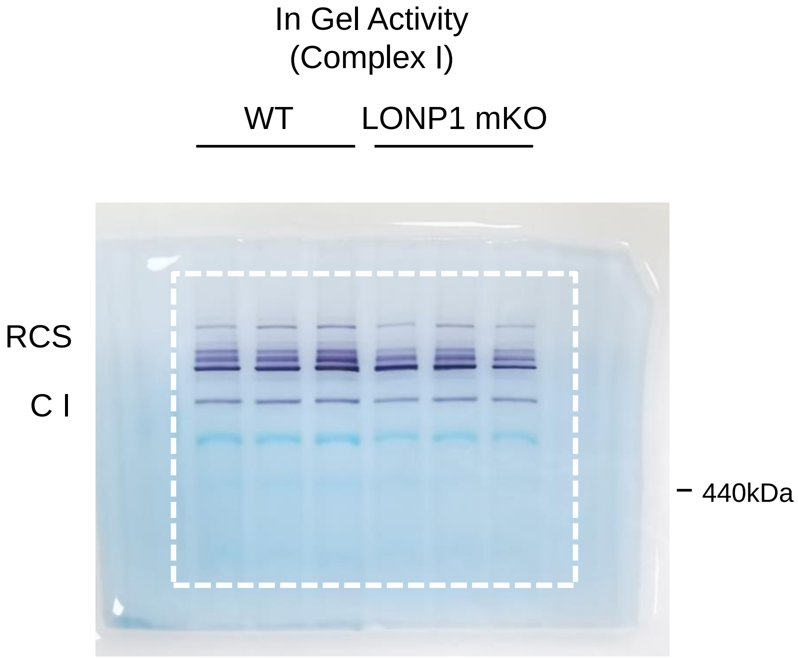

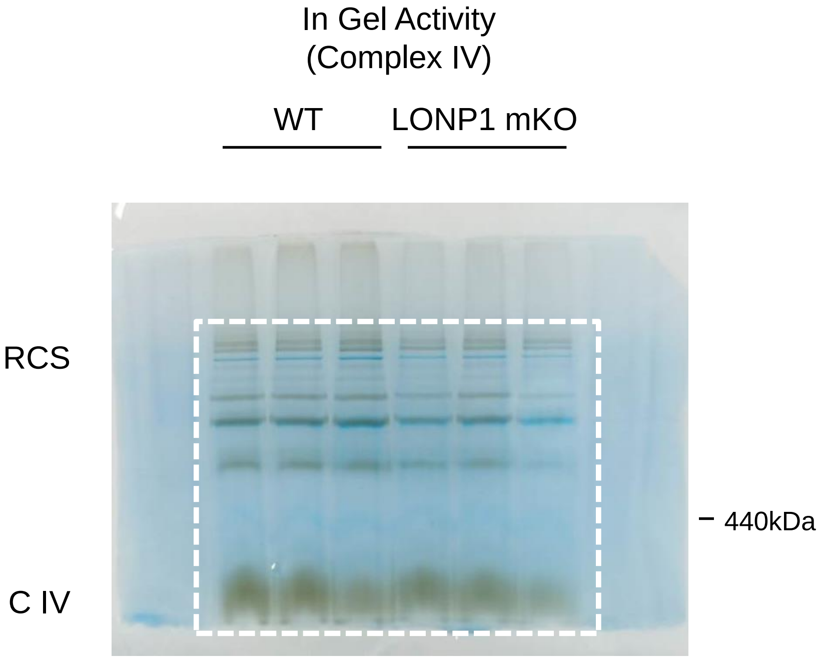

Uncropped scans for Supplementary Figure 5

Supplementary Figure 5a

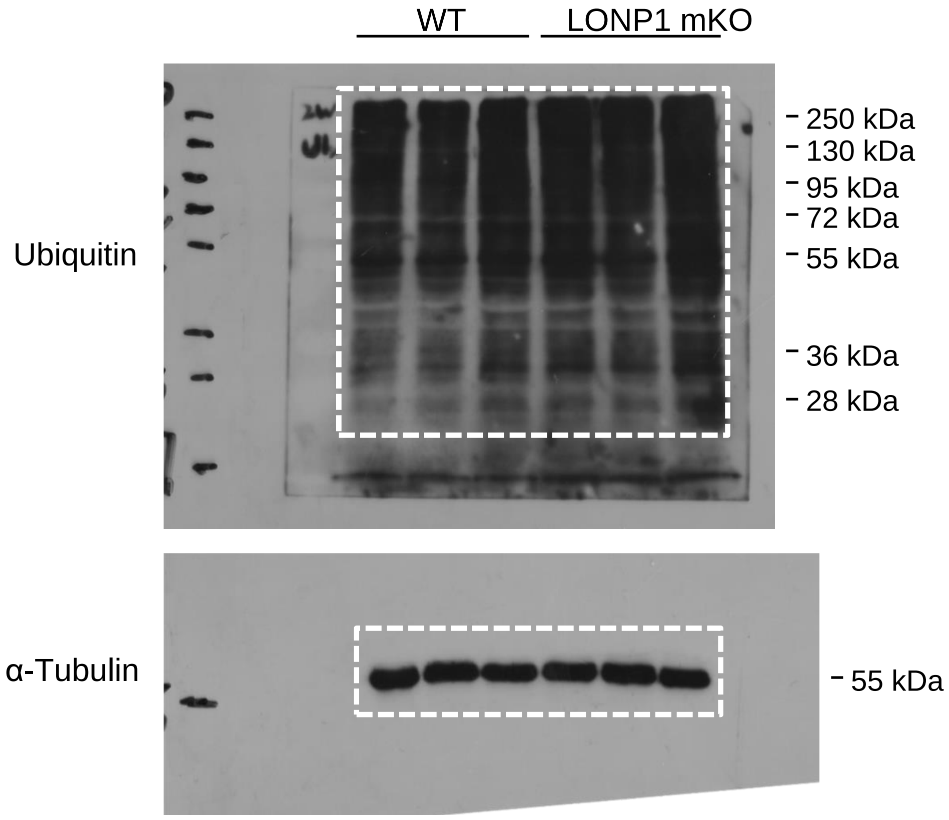

Supplementary Figure 5c

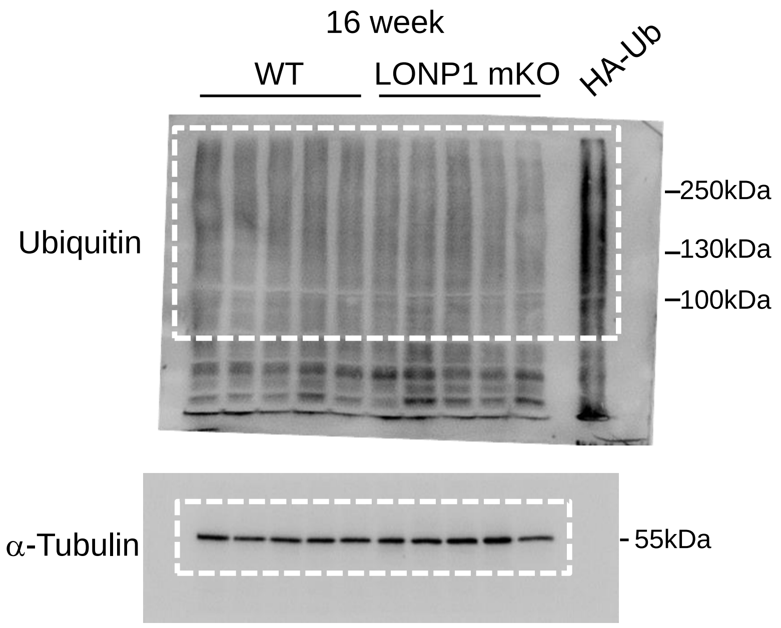

Supplementary Figure 5d

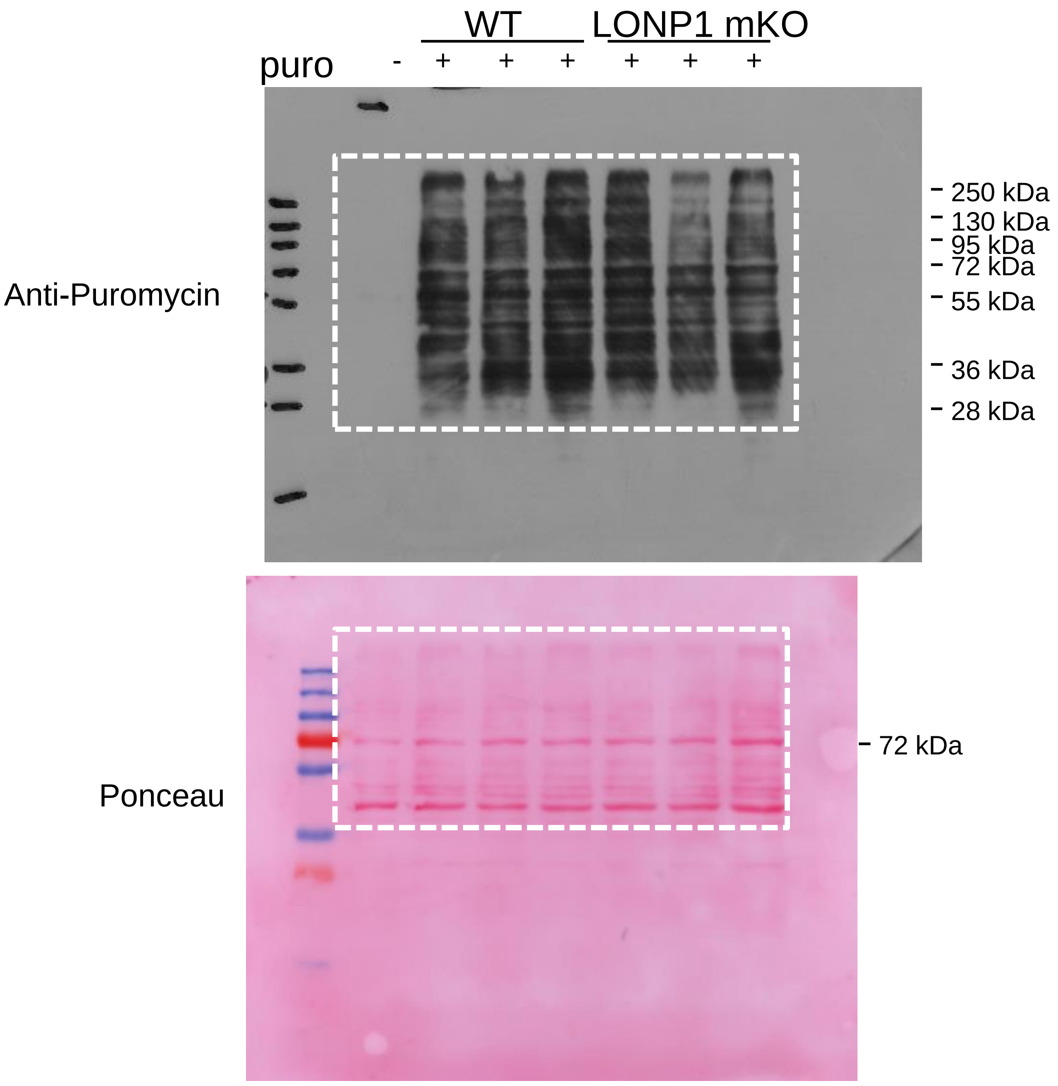

Supplementary Figure 5i

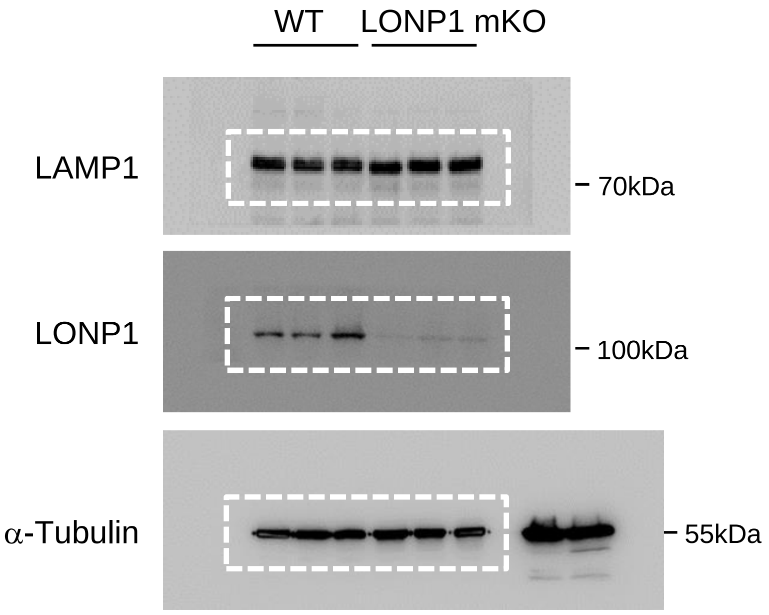

Uncropped scans for Supplementary Figure 6

Supplementary Figure 6b

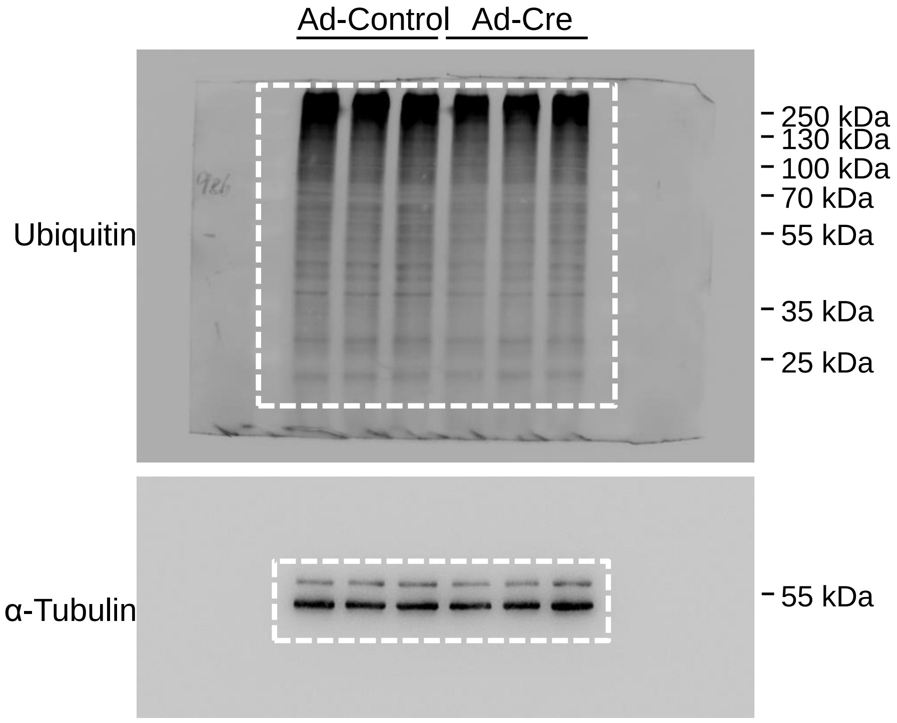

Supplementary Figure 6c

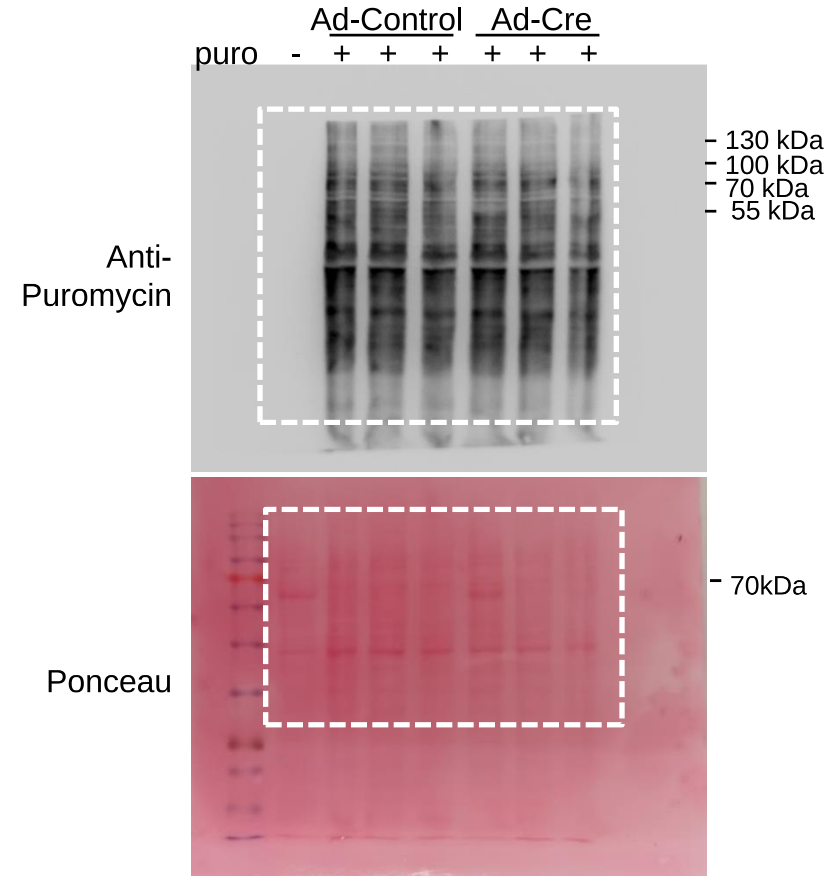

Supplementary Figure 6e

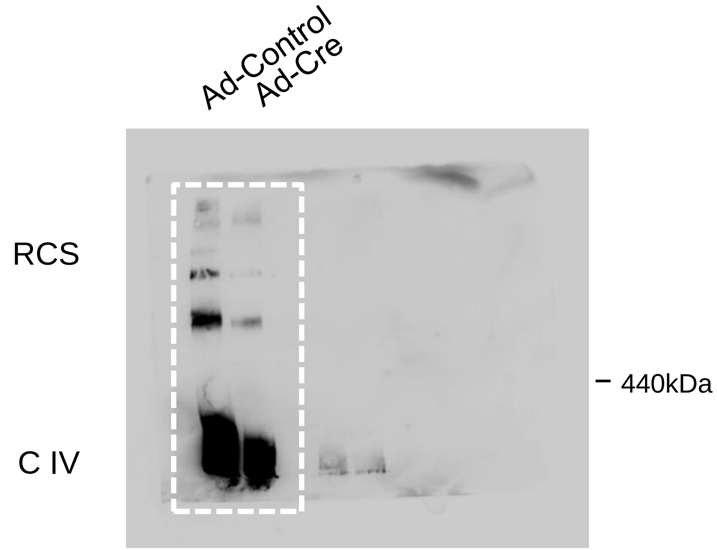

Supplementary Figure 6d

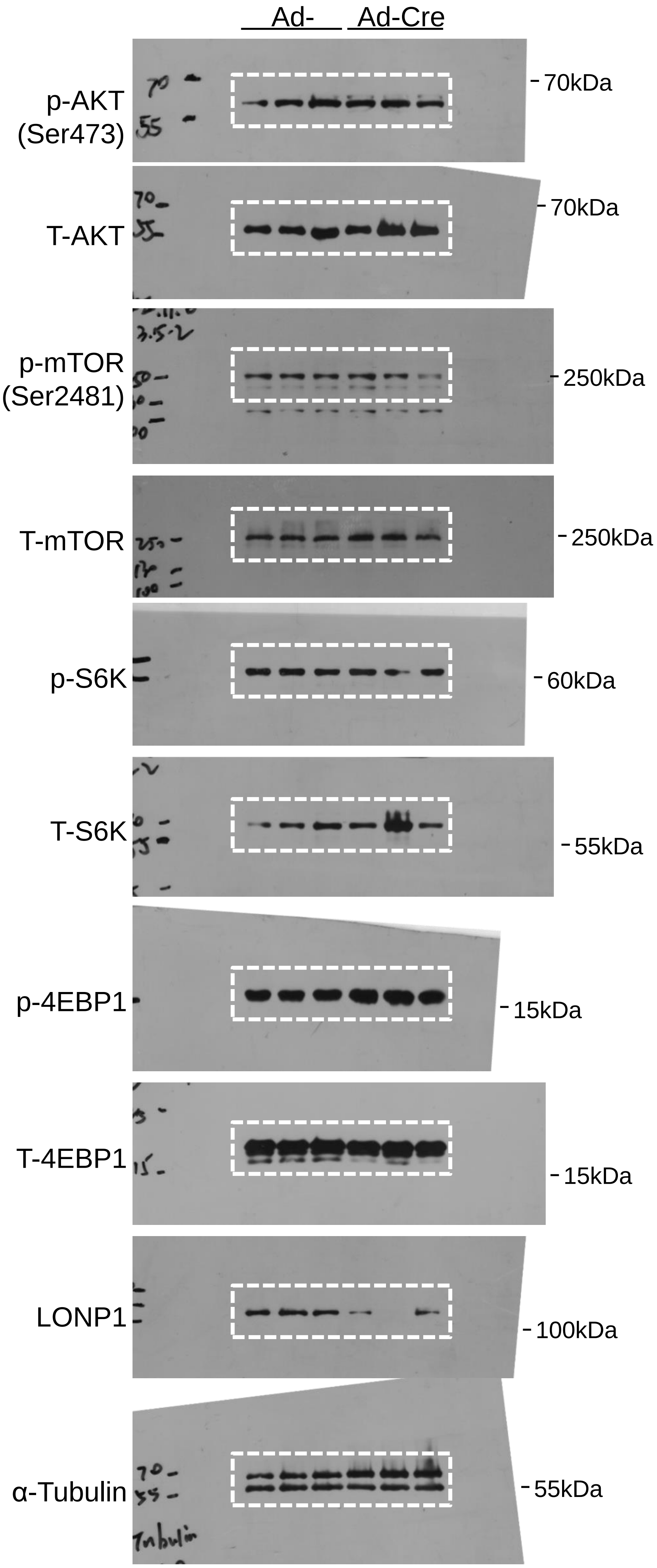

Uncropped scans for Supplementary Figure 7

Supplementary Figure 7b

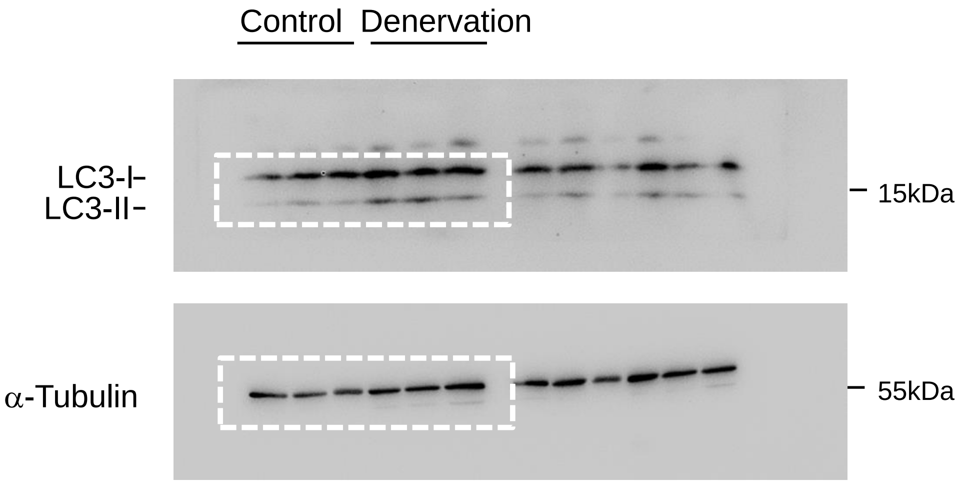

Uncropped scans for Supplementary Figure 8

Supplementary Figure 8a

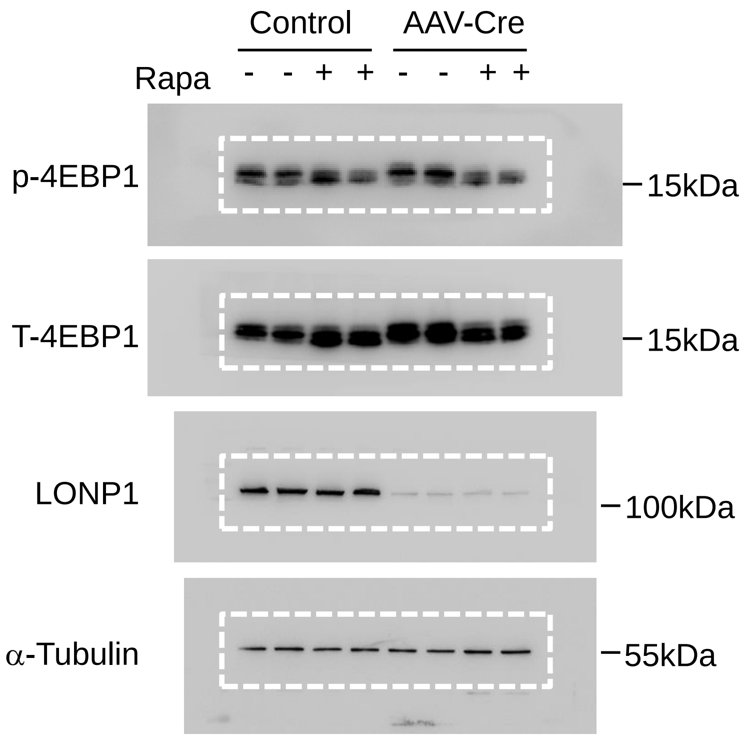

Uncropped scans for Supplementary Figure 9

Supplementary Figure 9b

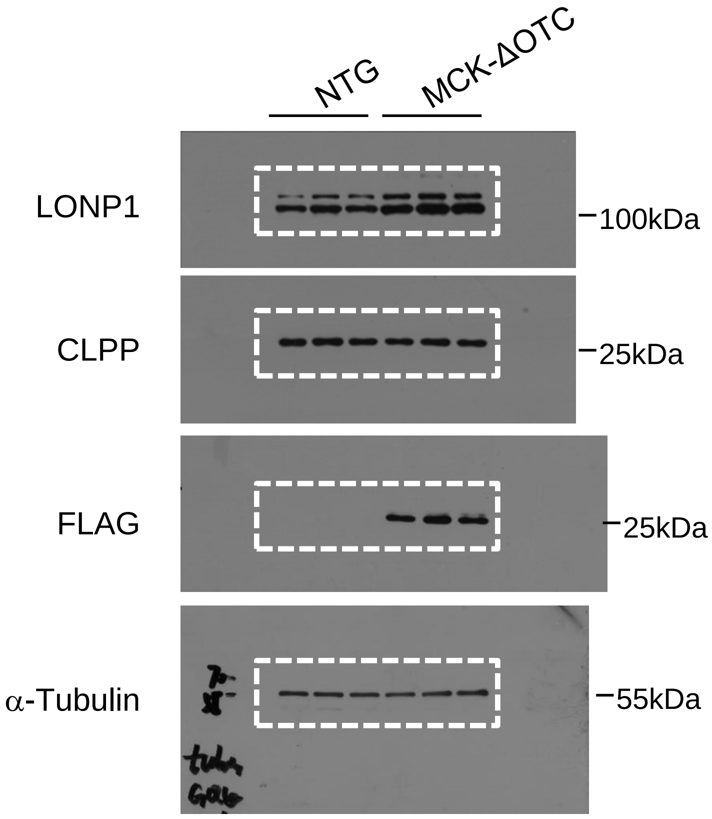

Supplementary Figure 9c

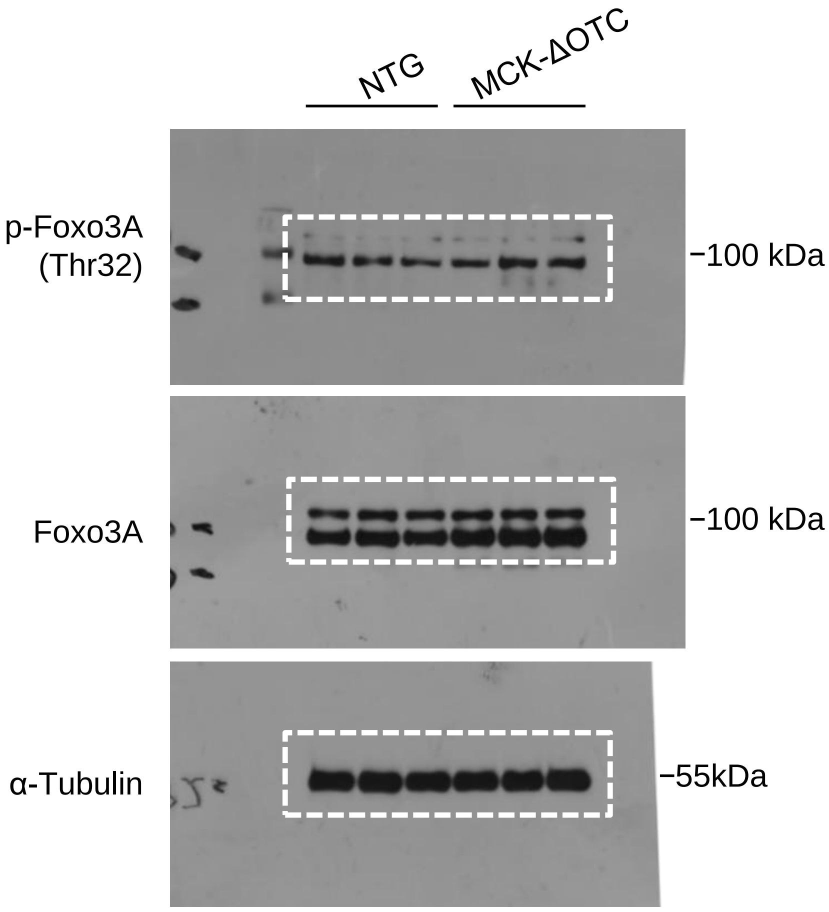

Supplement: Supplementary file 1 — Supplementary Information [file 41467_2022_28557_MOESM1_ESM.pdf]
