# Supplementary material for: Disuse-associated loss of the protease LONP1 in muscle impairs mitochondrial function and causes reduced skeletal muscle mass and strength
Source: Nat Commun. 2022 Feb 16;13:894. doi: 10.1038/s41467-022-28557-5 (PMC8850466; doi:10.1038/s41467-022-28557-5)
Supplement: Supplementary file 3 — Reporting Summary [file 41467_2022_28557_MOESM3_ESM.pdf]

nature research

Corresponding author(s): Zhenji Gan

Last updated by author(s): Jan 7, 2022

Reporting Summary

Nature Research wishes to improve the reproducibility of the work that we publish. This form provides structure for consistency and transparency in reporting. For further information on Nature Research policies, see our Editorial Policies and the Editorial Policy Checklist.

Statistics

For all statistical analyses, confirm that the following items are present in the figure legend, table legend, main text, or Methods section.

n/a	Confirmed	
☐	☒	The exact sample size (*n*) for each experimental group/condition, given as a discrete number and unit of measurement
☐	☒	A statement on whether measurements were taken from distinct samples or whether the same sample was measured repeatedly
☐	☒	The statistical test(s) used AND whether they are one- or two-sided<br>*Only common tests should be described solely by name; describe more complex techniques in the Methods section.*
☒	☐	A description of all covariates tested
☒	☐	A description of any assumptions or corrections, such as tests of normality and adjustment for multiple comparisons
☐	☒	A full description of the statistical parameters including central tendency (e.g. means) or other basic estimates (e.g. regression coefficient) AND variation (e.g. standard deviation) or associated estimates of uncertainty (e.g. confidence intervals)
☐	☒	For null hypothesis testing, the test statistic (e.g. *F*, *t*, *r*) with confidence intervals, effect sizes, degrees of freedom and *P* value noted<br>*Give P values as exact values whenever suitable.*
☒	☐	For Bayesian analysis, information on the choice of priors and Markov chain Monte Carlo settings
☒	☐	For hierarchical and complex designs, identification of the appropriate level for tests and full reporting of outcomes
☐	☒	Estimates of effect sizes (e.g. Cohen's *d*, Pearson's *r*), indicating how they were calculated

Our web collection on statistics for biologists contains articles on many of the points above.

Software and code

Policy information about availability of computer code

Data collection: mRNA-seq were performed using Illumina HiSeq 4000, and paired-end, 150 nt reads were obtained from the sequencing lane. MS data acquisition was performed by LC-MS/MS analysis using a TripleTOF 5600+ System (AB SCIEX, Concord, Ontario, Canada) coupled with a NanoLC.2D (Eksigent Technologies). The original MS data were submitted to ProteinPilot Software (version 4.5, AB SCIEX) with Paragon Algorithm (4.5.0.0 1654) for data analysis. MS/MS data were searched against Mus musculus in UniProt database (April 9, 2016, containing 160,566 sequences, http://www.uniprot.org/proteomes/UP000005640).

Data analysis: For mRNA-Seq, we used TopHat Version 2.0.14; Cufflinks Version 2.2.1 and GSEA (Gene Set Enrichment Analysis). The original MS data were submitted to ProteinPlot Software (version 4.5, AB SCIEX) with Paragon Algorithm (4.5.0.0 1654) for data analysis. Image analysis was performed with Image J (version 1.51, https://imagej. nih.gov/), Waterfall plot and heatmap analysis were performed by ggplot2 package in R software (Version 4.1.1) and Morpheus (https://software.broadinstitute.org/morpheus)

For manuscripts utilizing custom algorithms or software that are central to the research but not yet described in published literature, software must be made available to editors and reviewers. We strongly encourage code deposition in a community repository (e.g. GitHub). See the Nature Research guidelines for submitting code & software for further information.

Data

Policy information about availability of data

All manuscripts must include a data availability statement. This statement should provide the following information, where applicable:

- Accession codes, unique identifiers, or web links for publicly available datasets
- A list of figures that have associated raw data
- A description of any restrictions on data availability

Data availability. The muscle LONP1 mKO RNA-seq data generated in this study have been deposited in the NCBI Gene Expression Omnibus database under

accession number GSE166071 [https://www.ncbi.nlm.nih.gov/geo/query/acc.cgi?acc=GSE166071]. The proteomics data generated in this study have been deposited in PRIDE under accession code PXD029722 [https://www.ebi.ac.uk/pride/archive/projects/PXD029722]. MS/MS data were searched against Mus musculus in UniProt database (April 9, 2016, containing 160,566 sequences, http://www.uniprot.org/proteomes/UP000005640). All other data supporting the findings of this study are available with the article, and can also be obtained from the authors. Source data are provided with this paper. There is no restrictions on availability of data that support the finding of this study.

Field-specific reporting

Please select the one below that is the best fit for your research. If you are not sure, read the appropriate sections before making your selection.

☒ Life sciences ☐ Behavioural & social sciences ☐ Ecological, evolutionary & environmental sciences

For a reference copy of the document with all sections, see nature.com/documents/nr-reporting-summary-flat.pdf

Life sciences study design

All studies must disclose on these points even when the disclosure is negative.

Sample size	Sample sizes were indicated in the legend of each Figure and Supplementary Figure. No statistical tests were performed to pre-determine sample size. Sample sizes for the skeletal muscle studies were selected due to more than 10 years previous experience demonstrating the minimum number of animals necessary to achieve statistically significant and reproducible results (Gan Z et al. 2011. Genes Dev. PMID: 22135324; Gan Z et al. 2013. J Clin Invest. PMID: 23676496; Liu J et al. 2016. EMBO Mol Med. PMID:27506754; Fu T et al. 2018. Cell Rep. PMID: 29719250; Liu L et al. 2020. J Clin Invest. PMID: 32544095; Xiao L et al. 2021. Plos Genet. PMID: 33780446; He S et al. 2021. J Clin Invest. PMID: 34283807). For experiments involving cell cultures, at least 3 biological replicates per condition was used to enable statistical analysis.
Data exclusions	No data were excluded from the analysis.
Replication	The experimental findings were reliably reproduced, for representative data used for statistical analysis, the number of animals or experiments is described in corresponding figure legends.
Randomization	The muscle mass in LONP1 mKO mice were compared to WT littermate controls. For human supraspinatus muscle study, a previous study have reported a reproducible and reliable method to define supraspinatus muscle atrophy with the help of MRI (Thomazeau H et al., 1996 PMID: 8686465). Specifically, by calculating the occupation ratio which is the ratio between the surface of the cross-section of the muscle belly and that of the supraspinatus fossa, it allowed a reliable measurement of supraspinatus muscle atrophy, and the muscle with a ratio greater than 0.60 can be considered as normal or non-atrophied, while values below 0.60 suggest muscle atrophy. We thus evaluated the atrophy of the supraspinatus muscle using the MRI-based method. Briefly, MRI studies were carried out on all patients. The supraspinatus muscles were assessed using the most lateral oblique-sagittal section, which appears as “Y-shaped view” and we calculated the occupation ratio of the supraspinatus fossa by the muscle belly as previously described (Thomazeau H et al., 1996 PMID: 8686465). Details on subject characteristics are provided in Supplementary Table 1. Based on the occupation ratio, we divided the patients into two groups (non-atrophy group, mean ratio 0.76; atrophy group, mean ratio 0.50).
Blinding	In many applications (the fluorescence imaging, RNA-seq, and histological analysis, Mass spectrometry analysis, confocal microscopy, exercise stress test, muscle strength and tetanic contraction force measurements, X-ray, mitochondrial respiration studies, transmission electron microscopy. etc.), acquisition of the data was performed in a blinded fashion.<br>Investigators were not blinded during allocating animal experiment, because the investigators need to conduct genotyping PCRs at the age of 2 weeks for the mice, therefore investigators were not blinded for group identification or genotype identification for the mouse models used in this study. Blinding was not relevant to the other experiments in cells because the investigators needed to know what cell type they had to culture and process the cells by themselves.

Reporting for specific materials, systems and methods

We require information from authors about some types of materials, experimental systems and methods used in many studies. Here, indicate whether each material, system or method listed is relevant to your study. If you are not sure if a list item applies to your research, read the appropriate section before selecting a response.

Materials & experimental systems

n/a	Involved in the study	
☐	☒	Antibodies
☐	☒	Eukaryotic cell lines
☒	☐	Palaeontology and archaeology
☐	☒	Animals and other organisms
☐	☒	Human research participants
☒	☐	Clinical data
☒	☐	Dual use research of concern

Methods

n/a	Involved in the study	
☒	☐	ChIP-seq
☒	☐	Flow cytometry
☒	☐	MRI-based neuroimaging

Antibodies

Antibodies used

Antibodies directed against LONP1 (15440-1-AP, 1:2,500 dilution), CLPP (15698-1-AP, 1:2,500 dilution), NDUFB8 (14794-1-AP, 1:1,000 dilution), ND1 (19703-1-AP, :1,000 dilution), SDHA (14865-1-AP, 1:1,000 dilution), UQCRC2 (14742-1-AP, 1:1,000 dilution), COX4 (11242-1-AP, 1:1,000 dilution), ATP5A (14676-1-AP, 1:1,000 dilution), MFN1 (13798-1-AP, 1:1,000 dilution), DRP1 (12957-1-AP, 1:1,000 dilution), PARK7 (11681-1-AP, 1:1,000 dilution), PINK1 (23274-1-AP, 1:1,000 dilution), GAPDH (60004-1-Ig, 1:1,000 dilution) were all from Proteintech; antibodies directed against OPA1 (612606, 1:1,000 dilution) and Tim23 (#611222, 1:1,000 dilution) were from BD Biosciences; antibodies directed against a-tubulin (bs1699, 1:5,000 dilution) were from Bioworld; antibodies directed against GFP (sc-9996, 1:1,000 dilution), MFN2 (sc-100560, 1:500 dilution) and Ubiquitin (sc-8017, 1: 1,000 dilution) were from Santa Cruz; anti-LC3 antibody (NB100-2331, 1:1,000 dilution) and P62 (NBP1-48320, 1:1,000 dilution) were from Novus Biologicals; anti-Flag antibody (#F1804, 1: 1,000 dilution) and HA (H9658, 1:10,000 dilution) were from Sigma; anti-FOXO3A antibody (A0102, 1:1,000 dilution) was from Abconal, antibodies directed against p-FOXO3A Thr32 (#9464, 1:1,000 dilution), p-AKT Ser473 (#4060, 1:1,000 dilution), AKT (#9272, 1:1,000 dilution), p-mTOR Ser2448 (#5536, 1:1,000 dilution), p-mTOR Ser2481 (#2974, 1:1,000 dilution), mTOR (#2983, 1:1,000 dilution), p-S6K Thr389 (#9234, 1:1,000 dilution), S6K (#2708, 1:1,000 dilution), p-4EBP1 Thr37/46 (#2855, 1:1,000 dilution) and 4EBP1 (#9644, 1:1,000 dilution), LAMP1 (# 3243, 1:1,000 dilution), p-AMPKα Thr172 (#2535, 1:1,000 dilution) and AMPKα (#5831, 1:1,000 dilution) were from Cell Signaling Technology; anti-puromycin antibody (MABE343, clone 12D10, 1:5000 dilution) was from Sigma; anti-TFEB antibody (A303-673A, 1:200 dilution) was from Bethyl Laboratories; anti-MTCO1 antibody (ab14705, 1:1000 dilution) was from abcam; antibodies directed against MHC1 (BA-D5) and MHC2b (BF-F3) were purchased from the Developmental Studies Hybridoma Bank.
Antibodies are also listed in the Methods section under their respective experimental method.

Validation

All antibodies used in this study are commercial.
1. LONP1, reactivity: human, mouse, rat, application: IF, IHC, IP, WB, ELISA, manufacturer's website: https://www.ptgcn.com/products/LONP1-Antibody-15440-1-AP.htm
Moreover, the specificity of anti-LONP1 (15440-1-AP, 1:2,500 dilution) was further evaluated using WT and LONP1 mKO muscles in Supplementary Fig. 2b, Fig. 4e, 4h,4i. Specifically, we only detected a specific band corresponding to mouse LONP1 in WT but not in LONP1 mKO muscles by immunoblotting using the anti-LONP1 antibodies.
2. CLPP, reactivity: human, mouse, rat, application: IF, WB, manufacturer's website: https://www.ptgcn.com/products/CLPP-Antibody-15698-1-AP.htm
3. NDUFB8, reactivity: human, mouse, rat, application: IF, IHC, IP, WB, ELISA, manufacturer's website: https://www.ptgcn.com/products/NDUFB8-Antibody-14794-1-AP.htm
4. ND1, reactivity: human, mouse, application: IF, IHC, IP, WB, ELISA, manufacturer's website: https://www.ptgcn.com/products/ND1-Antibody-19703-1-AP.htm
5. SDHA, reactivity: human, mouse, rat, application: IF, IHC, IP, WB, ELISA, manufacturer's website: https://www.ptgcn.com/products/SDHA-Antibody-14865-1-AP.htm
6. UQCRC2, reactivity: human, mouse, rat, application: IF, IHC, IP, WB, ELISA, manufacturer's website: https://www.ptgcn.com/products/UQCRC2-Antibody-14742-1-AP.htm
7. COX4, reactivity: human, mouse, rat, application: IF, IHC, IP, WB, ELISA, manufacturer's website: https://www.ptgcn.com/products/COX4I1-Antibody-11242-1-AP.htm
8. ATP5A, reactivity: human, mouse, rat, application: IF, IHC, WB, ELISA, manufacturer's website: https://www.ptgcn.com/products/ATP5A1-Antibody-14676-1-AP.htm
9. MFN1, reactivity: human, mouse, rat, application: IHC, WB, ELISA, manufacturer's website: https://www.ptgcn.com/products/MFN1-Antibody-13798-1-AP.htm, KD/KO validated by Proteintech.
10. DRP1, reactivity: human, mouse, rat, application: FC, IF, IHC, IP, WB, ELISA, manufacturer's website: https://www.ptgcn.com/products/DNM1L,DLP1-Antibody-12957-1-AP.htm, KD/KO validated by Proteintech.
11. PARK7, reactivity: human, mouse, rat, application: IF, IHC, IP, WB, ELISA, manufacturer's website: https://www.ptgcn.com/products/PARK7,DJ-1-Antibody-11681-1-AP.htm, KD/KO validated by Proteintech and our results
12. PINK1, reactivity: human, mouse, application: IF, IHC, WB, ELISA, manufacturer's website: https://www.ptgcn.com/products/PINK1-Antibody-23274-1-AP.htm, KD/KO validated by Proteintech.
13. GAPDH, reactivity: human, mouse, rat, yeast, plant, zebrafish, application: FC, IF, IP, WB, ELISA, manufacturer's website: https://www.ptgcn.com/products/GAPDH-Antibody-60004-1-Ig.htm
14. OPA1, reactivity: Human (QC Testing), Mouse, Rat, Dog, Chicken (Tested in Development), application: WB, IF, manufacturer's website: https://www.bdbiosciences.com/en-us/search-results?searchKey=OPA1
15. TIM23, reactivity: Mouse (QC Testing), Human, Rat (Tested in Development), application: WB (Routinely Tested), IF (Tested during Development), manufacturer's website: https://www.bdbiosciences.com/en-us/products/reagents/microscopy-imaging-reagents/immunofluorescence-reagents/purified-mouse-anti-tim23.611222
16. Tubulin: reactivity: human, mouse, rat, application: IHC, WB, IF, manufacturer's website: https://www.bioworlde.com/index.php?c=product&a=search&keyword=bs1699
17. GFP, application: WB, IP, IF, FCM, ELISA, manufacturer's website: https://www.scbt.com/p/gfp-antibody-b-2?requestFrom=search
18. MFN2, reactivity: human, mouse, rat, application: IF, IHC, IP,WB, ELISA, manufacturer's website: https://www.scbt.com/zh/p/mfn2-antibody-xx-1?requestFrom=search
19. Ubiquitin, reactivity: human, mouse, rat, drosophila, application: IHC, WB, ELISA, manufacturer's website: https://www.scbt.com/p/ubiquitin-antibody-p4d1?requestFrom=search
20. LC3, reactivity: human, mouse, rat, amphibian, canine, fish, plant, zebrafish, bovine, application: WB, CHIP, ELISA, FLOW, IF, IHC, manufacturer's website: https://www.novusbio.com/products/lc3a-antibody_nb100-2331
21. P62, reactivity: human, mouse, rat, chicken, porcine, canine, application: WB, FLOW, IF, IHC, manufacturer's website: https://www.novusbio.com/products/p62-sqstm1-antibody_nbp1-48320
22. FLAG, application: WB, IP, IHC, IF, manufacturer's website: https://www.sigmaaldrich.cn/CN/zh/product/sigma/f1804
23. HA, reactivity: mouse, application: WB, IP, ICC, ELISA, manufacturer's website: https://www.sigmaaldrich.cn/CN/en/product/sigma/h9658
24. FOXO3A, reactivity: human, mouse, rat, application WB, IHC, IF, IP, ChIP, manufacturer's website: https://abclonal.com.cn/

catalog/A0102
25. P-FOXO3A Thr32, reactivity: human, mouse, rat, monkey, application: WB, IHC, IF, IP, ChIP, F, E-P, manufacturer's website: https://www.cellsignal.cn/products/primary-antibodies/phospho-foxo1-thr24-foxo3a-thr32-antibody/9464?site-search-type=Products&N=4294956287&Ntt=%239464&fromPage=plp&_requestid=1886172
26. p-AKT Ser473, reactivity: human, mouse, rabbit, hamster, monkey, D. melanogaster, zebra fish, bovine, application: WB, IP, IHC, IF, F, manufacturer's website: https://www.cellsignal.cn/products/primary-antibodies/phospho-akt-ser473-d9e-xp-rabbit-mab/4060?site-searchtype=Products&N=4294956287&Ntt=%234060&fromPage=plp&_requestid=1887076
27. AKT, reactivity: human, mouse, rabbit, monkey, hamster, chicken, D. melanogaster, zebrafish, bovine, dog, pig, application: WB, IP, IHC, IF, F, manufacturer's website: https://www.cellsignal.com/products/primary-antibodies/akt-antibody/9272?site-search-type=Products&N=4294956287&Ntt=%239272&fromPage=plp&_requestid=1887544&country=USA
28. p-mTOR Ser2448, reactivity: human, mouse, rabbit, monkey, application: WB, IP, IF manufacturer's website: https://www.cellsignal.com/products/primary-antibodies/phospho-mtor-ser2448-d9c2-xp-rabbit-mab/5536?site-search-type=Products&N=4294956287&Ntt=%235536&fromPage=plp&_requestid=1888762
29. p-mTOR Ser2481, reactivity: human, mouse, rabbit, monkey, application: WB, manufacturer's website: https://www.cellsignal.com/products/primary-antibodies/phospho-mtor-ser2481-antibody/2974?site-search-type=Products&N=4294956287&Ntt=%232974&fromPage=plp&_requestid=1889354
30. mTOR, reactivity: human, mouse, rabbit, monkey, application: WB, IP, IHC, IF, F, manufacturer's website: https://www.cellsignal.com/products/primary-antibodies/mtor-7c10-rabbit-mab/2983?site-search-type=Products&N=4294956287&Ntt=%232983&fromPage=plp&_requestid=1869546
31. p-S6K Thr389, reactivity: human, mouse, rabbit, monkey, application: WB, manufacturer's website: https://www.cellsignal.com/products/primary-antibodies/phospho-p70-s6-kinase-thr389-108d2-rabbit-mab/9234?site-search-type=Products&N=4294956287&Ntt=%239234&fromPage=plp
32. S6K, reactivity: human, application: WB, manufacturer's website: https://www.cellsignal.com/products/primary-antibodies/p70-s6-kinase-49d7-rabbit-mab/2708?site-search-type=Products&N=4294956287&Ntt=%232708&fromPage=plp
33. p-4EBP1 Thr37/46, reactivity: human, mouse, rabbit, monkey, D. melanogaster, application: WB, IHC, IF, F, manufacturer's website: https://www.cellsignal.com/products/primary-antibodies/phospho-4e-bp1-thr37-46-236b4-rabbit-mab/2855?site-search-type=Products&N=4294956287&Ntt=%232855%2C&fromPage=plp&_requestid=1870112
34. 4EBP1, reactivity: WB, IP, IHC, IF, F, application: human, mouse, rabbit, monkey, manufacturer's website: https://www.cellsignal.com/products/primary-antibodies/4e-bp1-53h11-rabbit-mab/9644?site-search-type=Products&N=4294956287&Ntt=%239644&fromPage=plp&_requestid=1870403
35. LAMP1, reactivity: human, mouse, monkey, application: WB, manufacturer's website: https://www.cellsignal.com/products/primary-antibodies/lamp1-c54h11-rabbit-mab/3243?site-search-type=Products&N=4294956287&Ntt=%23+3243&fromPage=plp&_requestid=1870582
36. p-AMPKα Thr172, reactivity: Human, Mouse, Rabbit, Monkey, Hamster, D. melanogaster, S. cerevisiae, application: WB, IP, IHC, manufacturer's website: https://www.cellsignal.com/products/primary-antibodies/phospho-ampka-thr172-40h9-rabbit-mab/2535?site-search-type=Products&N=4294956287&Ntt=%232535&fromPage=plp&_requestid=1870728
37. AMPKα, reactivity: Human, Mouse, Rabbit, Monkey, Bovine, application: WB, IP, manufacturer's website: https://www.cellsignal.com/products/primary-antibodies/ampka-d5a2-rabbit-mab/5831?site-searchtype=Products&N=4294956287&Ntt=%235831&fromPage=plp&_requestid=1871041
38. Puromycin antibody, reactivity: all, application: FLOW, IHC, IF, IP, WB, manufacturer's website: https://www.sigmaaldrich.cn/CN/zh/product/mm/mabe343
39. TFEB, reactivity: human, mouse, application: WB, IP, IHC, manufacturer's website: https://www.biomol.com/products/antibodies/primary-antibodies/general/anti-tfeb-a303-673a-t?number=A303-673A
40. MTCO1, reactivity: human, mouse, cow, rat, application: IHC, WB, FLOW, manufacturer's website: https://www.abcam.cn/mtco1-antibody-1d6e1a8-ab14705.html
41. MHC1(BA-D5): reactivity: bovine, canine, fish, goat, guinea pig, horse, human, lamb, llama, mouse, porcine, rabbit, rat, zebrafish, application: IHC, WB, IF, manufacturer's website: https://dshb.biology.uiowa.edu/BA-D5
42. MHC2b(BF-F3): reactivity: bovine, mouse, porcine, rat, sheep, application: IHC, WB, IF, ELISA, manufacturer's website: https://dshb.biology.uiowa.edu/BF-F3

Eukaryotic cell lines

Policy information about cell lines

Cell line source(s)	HEK293T Cells were obtained from the American Type Culture Collection and were cultured at 37°C and 5% CO2 in Dulbecco's modified Eagle's medium (DMEM) supplemented with 10% FBS, 1,000 U/ml penicillin and 100 g /ml streptomycin.
Authentication	The cell line was not authenticated.
Mycoplasma contamination	Mycoplasma contamination was not tested in the study.
Commonly misidentified lines (See ICLAC register)	No commercial misidentified cells were used.

Animals and other organisms

Policy information about studies involving animals; ARRIVE guidelines recommended for reporting animal research

Laboratory animals	All animal studies were conducted in strict accordance with the institutional guidelines for the humane treatment of animals and were approved by the IACUC committees at the Model Animal Research Center (MARC) of Nanjing University (Approval No. GZJ07). Male C57BL/6J wild-type mice were from GemPharmatech Co., Ltd (Jiangsu, China). The LONP1 mKO, mt-Keima Tg, and CAG-CAT-

	MitoTimer reporter mice were back-crossed to the C57BL/6J background for more than 6 generations. MCK-ΔOTC transgenic mice were in C57BL/6J background. Male mice between 2 and 40 weeks of age were used for all experiments. The mice were maintained with free access to pellet food and water in plastic cages at 21 ± 2 °C and kept on a 12 h light–dark cycle. The further details can be found the Animal studies section.
Wild animals	No
Field-collected samples	No
Ethics oversight	The animal use and the experimental protocols were reviewed and approved by the IACUC committees at the Model Animal Research Center (MARC) of Nanjing University (Approval No: GZJ07).

Note that full information on the approval of the study protocol must also be provided in the manuscript.

Human research participants

Policy information about studies involving human research participants

Population characteristics	Human supraspinatus muscle samples were collected from 9 rotator cuff tear patients. Details on subject characteristics are provided in Supplementary Table 1.
Recruitment	Clinical ethical approval (study number 2021ky-001) in comply with the Declaration of Helsinkink1 was obtained from the Ethical Committee of Subei People's Hospital of Jiangsu Province, Yangzhou, China. A total of 9 patients with rotator cuff tear were recruited for this study voluntarily. Details of the research project and biopsy collection were explained to all subjects before entering into the study. Informed consent was obtained from all subjects. A previous study has reported a reproducible and reliable method to define supraspinatus muscle atrophy with the help of MRI (Thomazeau H et al. 1996. PMID: 8686465). Specifically, by calculating the occupation ratio which is the ratio between the surface of the cross-section of the muscle belly and that of the supraspinatus fossa, it allowed a reliable measurement of supraspinatus muscle atrophy, and the muscle with a ratio greater than 0.60 can be considered as normal or non-atrophied, while values below 0.60 suggest muscle atrophy. We thus evaluated the atrophy of the supraspinatus muscle using the MRI-based method. Briefly, MRI studies were carried out on all patients. The supraspinatus muscles were assessed using the most lateral oblique-sagittal section, which appears as “Y-shaped view” and we calculated the occupation ratio of the supraspinatus fossa by the muscle belly as previously described (Thomazeau H et al. 1996. PMID: 8686465). Details on subject characteristics are provided in Supplementary Table 1. Based on the occupation ratio, we divided the patients into two groups (non-atrophy group, mean ratio 0.76; atrophy group, mean ratio 0.50).
Ethics oversight	The Ethical Committee of Subei People's Hospital of Jiangsu Province (Clinical ethical approval: 2021ky-001), Yangzhou, China.

Note that full information on the approval of the study protocol must also be provided in the manuscript.
